# Angelman Syndrome and Angelman-like Syndromes Share the Same Calcium-Related Gene Signatures

**DOI:** 10.3390/ijms22189870

**Published:** 2021-09-13

**Authors:** Julia Panov, Hanoch Kaphzan

**Affiliations:** Laboratory for Neurobiology of Psychiatric Disorders, Sagol Department of Neurobiology, University of Haifa, Haifa 3498838, Israel; juliapanov.uni@gmail.com

**Keywords:** Angelman syndrome, Angelman-like syndromes, calcium signaling, RNA sequencing, transcriptome, calcium-target genes, calcium-regulating genes

## Abstract

Angelman-like syndromes are a group of neurodevelopmental disorders that entail clinical presentation similar to Angelman Syndrome (AS). In our previous study, we showed that calcium signaling is disrupted in AS, and we identified calcium-target and calcium-regulating gene signatures that are able to differentiate between AS and their controls in different models. In the herein study, we evaluated these sets of calcium-target and calcium-regulating genes as signatures of AS-like and non-AS-like syndromes. We collected a number of RNA-seq datasets of various AS-like and non-AS-like syndromes and performed Principle Component Analysis (PCA) separately on the two sets of signature genes to visualize the distribution of samples on the PC1–PC2 plane. In addition to the evaluation of calcium signature genes, we performed differential gene expression analyses to identify calcium-related genes dysregulated in each of the studied syndromes. These analyses showed that the calcium-target and calcium-regulating signatures differentiate well between AS-like syndromes and their controls. However, in spite of the fact that many of the non-AS-like syndromes have multiple differentially expressed calcium-related genes, the calcium signatures were not efficient classifiers for non-AS-like neurodevelopmental disorders. These results show that features based on clinical presentation are reflected in signatures derived from bioinformatics analyses and suggest the use of bioinformatics as a tool for classification.

## 1. Introduction 

Angelman syndrome (AS) is a rare genetic neurodevelopmental disorder, manifested by a happy demeanor, severe cognitive deficits, absence of speech, susceptibility for epilepsy, and motor impairments [1,2,3,4,5]. Its prevalence is between 1:10,000–1:20,000 [2,6]. The cause for AS is the loss of function of the *UBE3A* protein in the brain. In neurons, *UBE3A* is an imprinted gene, and only the maternal gene is expressed, while the paternal gene is silenced. In ~70% of AS cases, the loss of *UBE3A* is due to the deletion of small portions of the maternal chromosome 15(q11-13), which contains the *UBE3A* gene [7,8,9,10]. Other causes of AS include unipaternal disomy, imprinting defects, and *UBE3A* point mutations in the maternal gene [6,11,12]. Interestingly, in about 10% of the cases, the clinical phenotype is very similar to AS; however, none of the genetic screening tests point to the above-mentioned issues, and no other genetic neurodevelopmental disorder with similar clinical manifestation is diagnosed. 

In previous studies, we observed that activity-dependent calcium signaling is impaired in AS hippocampal pyramidal neurons [13]. This finding led us to investigate the expression profiles of calcium-related genes in AS [14]. We used the calcium genes database (CaDeGB) [15] to identify the calcium-related genes. These genes were divided into two categories according to their role in calcium signaling, calcium-target genes that are downstream genes affected by calcium signaling, and calcium-regulating genes that are upstream and modify calcium signaling. In our previous study [14], we found a list of calcium-target genes and a list of calcium-regulating genes that can serve as transcriptomic signatures for AS, distinguishing between AS mice and their healthy control littermates. Following this finding, we further demonstrated that these calcium-target and calcium-regulating genes can also serve as signatures for AS in two independent RNA sequencing datasets generated from two completely different human cellular models of AS [14]. Furthermore, we showed that other lists of randomly picked calcium-related genes were not able to serve as signatures for AS, making these found gene signatures distinctive. These results propelled us to examine these calcium-target and calcium-regulating gene signatures in a wide array of neurodevelopmental disorders, using publicly available transcriptome datasets extracted from various AS-like and non-AS-like disease models. 

For the first time, we show here that the calcium-related gene signatures for AS work also as signatures for additional syndromes that are clinically termed as AS-like syndromes or that have sufficient overlap of clinical symptoms with AS. However, they do not work as signatures for syndromes that are clinically different from AS. Based on the calcium-target and calcium-regulating signature gene lists, we presented each dataset of a distinct neurodevelopmental disorder utilizing a dimensionality-reduction approach, thus demonstrating the separation of each AS-like syndrome from its healthy controls. 

The significance of the herein reported results is two-fold. One, these results that are based on a bioinformatics analyses might serve as a basis for understanding the molecular pathophysiological differences between distinct neurodevelopmental disorders. Two, the herein findings suggest a novel method of developing a deeper diagnostic approach, which might alter classification methods of neurodevelopmental disorders. 

## 2. Results

Our aim was to determine whether the distinct signatures of calcium-target and calcium-regulating genes that we have previously found for Angelman Syndrome (AS) [14] are shared by other neurodevelopmental syndromes and whether there is a difference between AS-like and non-AS-like syndromes. In order to examine that, we re-analyzed publicly available transcriptome datasets of neurodevelopmental disorders in light of calcium-associated genes. The evaluated syndromes were divided into two groups—AS-like and non-AS-like—based on their clinical appearance [16]. 

**Figure 1:**
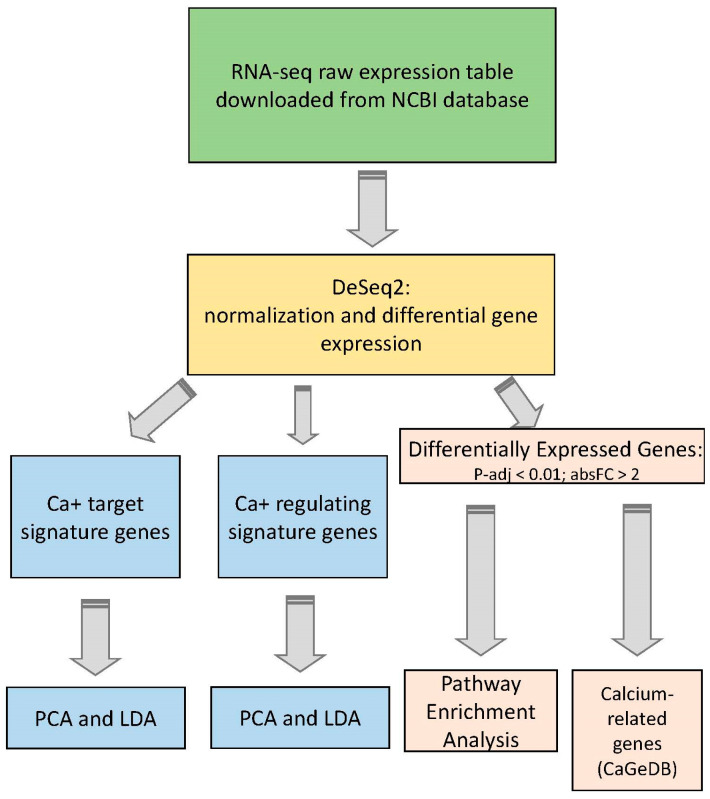
A schematic presentation of analysis steps for each dataset.

The schematic representation of the analysis steps for each dataset is presented in Figure 1.

For each studied syndrome, we obtained the raw expression matrix from the NCBI database (for full list of all datasets used in the study please refer to Table 1). 

The raw expression matrix was normalized using a normalization algorithm implemented in the DESeq2 R package [17]. After normalization, calcium-target and calcium-regulating gene signatures found in our study of calcium-associated genes in AS [14] were extracted, and a Principle Component Analysis (PCA) was performed using these two signatures separately. The PCA method [18,19] compresses the multidimensional distribution of points in a space of a smaller dimension (typically two-dimensional space) with minimal distortion of inter-point distances. PCA analysis gave the position of samples from each dataset on the two-dimensional plane keeping the distance between samples similar to the distance based on all original dimensions, i.e., the expression of calcium-target or calcium-regulating genes. The distribution of samples on the PC1–PC2 planes was used to visualize the difference between control and disease samples and to identify whether calcium-target and calcium-regulating signatures can be used to correctly classify the studied disorder. In addition, for each disorder, we identified differentially expressed genes using the DeSeq2 algorithm [17]. Enrichment analysis of Gene Ontology (GO) terms and biological pathways was performed using the DAVID Bioinformatics Recourses tool [20,21]. Utilizing the CaGeDB database [15] of calcium-associated genes, we extracted differentially expressed genes that are known to be either calcium-target or calcium-regulating. 

### 2.1. AS Like Syndromes

#### 2.1.1. Rett Syndrome (GSE105045, GSE128380)

Rett syndrome (RTT) is considered an AS-like syndrome and shares many clinical features with AS [16]. Approximately 95% of RTT cases are due to mutations in the *MECP2* gene, which is located on the X chromosome [22]. Females partially compensate for the loss of *MECP2* function with an extra intact copy on the homologous X chromosome, but this is not the case for males. Consequently, males have a severe phenotype and represent less than 1% of RTT patients. RTT occurs in 1 in 10–15,000 live female births, which makes it one of the most common causes of monogenic intellectual disability in females [23]. *MECP2* is an X-linked gene that recognizes DNA, and histone methylation marks and modifies the transcription [23]. *MECP2* is expressed in all tissues in the body with major expression in the central nervous system (CNS). 

We utilized two different RNA sequencing datasets for our study with a focus on the calcium-target and calcium-regulating gene expressions. One dataset is an mRNA sequencing of cerebellum of Mecp2 knockdown mice and their wild-type littermates [24]. Another dataset is a total RNA sequencing data generated from postmortem cingulate and temporal cortices of RTT patients and healthy controls [25]. 

**Figure 2:**
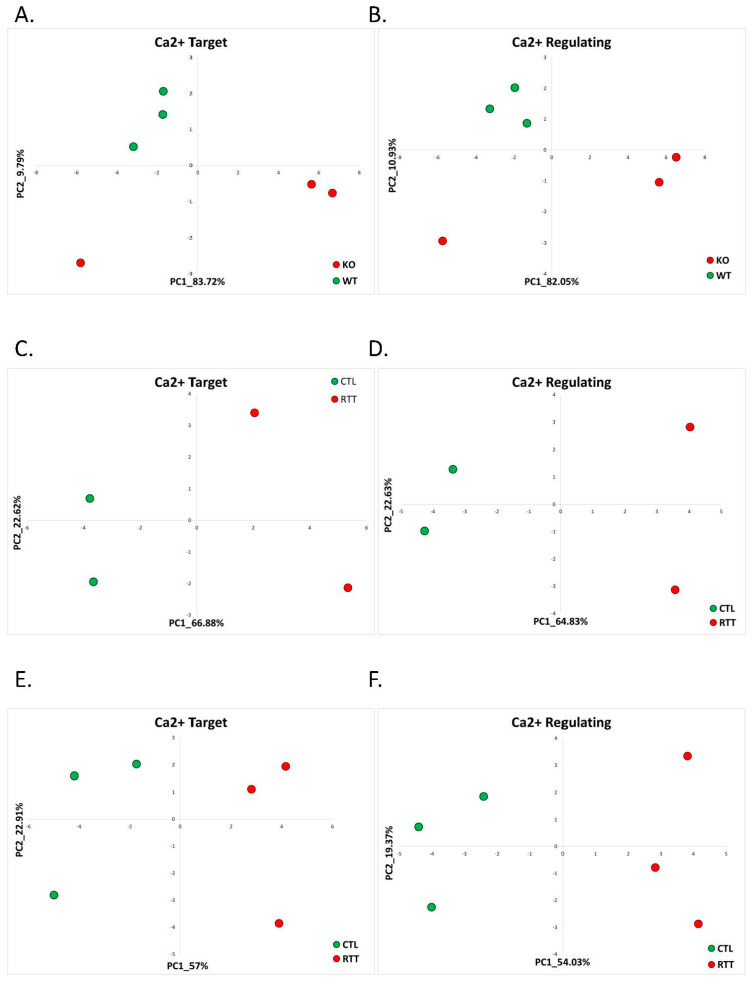
Rett Syndrome: ca-target and ca-regulating signatures. (**A**) PCA based on the expression of calcium-target signature genes in cerebellum of Mecp2-KO and WT mice. The WT samples are denoted in green, while the Mecp2-KO samples are in red. (**B**) PCA based on the expression of calcium-regulating signature genes in cerebellum of Mecp2-KO and WT mice. The WT samples are denoted in green, while the Mecp2-KO samples are in red. (**C**) PCA based on the expression of calcium-target signature genes in postmortem cingulate cortex of Rett patients and healthy controls. The control samples are denoted in green, while the Rett samples are in red. (**D**) PCA based on the expression of calcium-regulating signature genes in the postmortem cingulate cortex of Rett patients and healthy controls. The control samples are denoted in green, while the Rett samples are in red. (**E**) PCA based on the expression of calcium-target signature genes in the postmortem temporal cortex of Rett patients and healthy controls. The control samples are denoted in green, while the Rett samples are in red. (**F**) PCA based on the expression of calcium-regulating signature genes in the postmortem temporal cortex of Rett patients and healthy controls. The control samples are denoted in green, while the Rett samples are in red.

To determine whether our previously identified calcium-target and calcium-regulating signatures of AS can also discriminate Rett syndrome patients, we first studied the dataset generated from cerebellum of Mecp2 knockdown male mice (Mecp2-KO) and their wild-type (WT) controls available in the NCBI database under the accession number GSE105045 [24]. We found that in the mouse Rett model (Mecp2-KO mice), the calcium-target and the calcium-regulating signatures found in AS were good classifiers of Rett syndrome. The calcium-target and calcium-regulating signature genes separated the Rett syndrome samples from the healthy control samples in the PC1–PC2 space well (Figure 2A,B). Unsupervised analysis of differentially expressed genes in the mouse model of Rett syndrome did not reveal significantly enriched calcium-related pathways (Supplementary Figure S1A,B); however, it has been previously shown that calcium signaling is implicated in RTT pathology [26,27]. Focusing on the calcium-related differentially expressed genes utilizing the CaDeGB database, we found that in Mecp2-KO mice, 12.5% (four genes) of all differentially expressed genes were calcium-related (Table 2, Supplementary Table S1). Interestingly, all of the calcium-related genes were downregulated in cerebellum of Mecp2-KO compared to WT control mice.

To further examine whether AS-found calcium-target and calcium-regulating signatures are indicative of Rett syndrome, we utilized a publicly available human postmortem patient brain dataset. This dataset is an RNA sequencing generated from postmortem brain tissue samples of four female patients clinically diagnosed with Rett syndrome and four age-matched female donors (GSE128380) [25]. The etiology of two out of three patients is known and includes one patient with an *MECP2* mutation c473 C>T and another patient with the deletion of exon 1 of the *MECP2* gene. The etiology of the third patient is not known. The dataset contains transcriptomes from two brain regions of each individual: temporal and cingulate cortices. Following the authors’ finding of a significant 3′ bias in some of the samples, we excluded these samples from further analysis [25]. 

In both cingulate and temporal cortices of the Rett patients, the calcium-target and calcium-regulating signatures separated the Rett samples from healthy controls on the PC1–PC2 plane well (Figure 2C–F). Analyses of differentially expressed genes in both postmortem cingulate and temporal cortices of Rett syndrome patients revealed the enrichment of calcium-related gene ontology (GO) terms (Supplementary Figure S1C–F). In addition, we found that of all differentially expressed genes, 14.35% (86 genes) in the cingulate cortex and 13.31% (177 genes) in the temporal cortex were calcium-related (Table 2, Supplementary Tables S2–S3). 

#### 2.1.2. Pitt-Hopkins (GSE79663)

Another neurodevelopmental disorder with clinical presentation similar to Angelman syndrome is Pitt-Hopkins syndrome (PTHS). Similarly to AS, Pitt-Hopkins is associated with development delay, hypotonia and delays in motor development, severe language impairment, epilepsy, and autism [28]. PTHS is caused by haploinsufficiency of the TCF4 protein [29]. TCF4 is a transcription factor that binds the E-Box DNA element (5′-CANNTG-3′) and affects chromatin remodeling and transcription through the recruitment of histone acetyltransferases. TCF4 has been shown to affect neurodevelopment and play an important role in cognition, being associated with both schizophrenia and autism-spectrum intellectual disability in addition to PTHS [28]. Calcium signaling has been shown to be disrupted in mouse models of Tcf4 haploinsufficiency [28].

In order to determine whether our AS-found calcium-target and calcium-regulating signatures are also classifiers for PTHS, we re-analyzed the publicly available RNA sequencing dataset of dorsal telencephalons of Tcf4-knockout, Tcf4-heterozygous and WT mice at P0 (immediately after birth) (GSE79663) [30]. Studying the telencephalons of these Tcf4-knockout and heterozygous mice gives an indication of molecular disruptions in the cerebral cortex after initial development as projection neurons are generated in the proliferative ventricular zone and the subventricular zone of the embryonic telencephalon [30].

**Figure 3:**
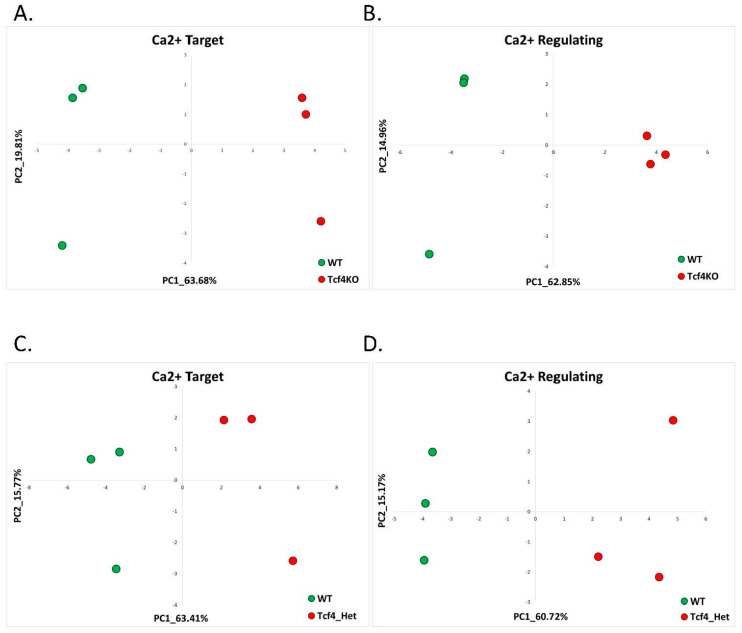
Pitt-Hopkins: ca-target and ca-regulating signatures. (**A**) PCA based on the expression of calcium-target signature genes in dorsal telencephalons of Tcf4 Knockout (Tcf4-KO) compared to control (WT) mice. The control samples are denoted in green, while the Tcf4 Knockout samples are in red. (**B**) PCA based on the expression of calcium-regulating signature genes in dorsal telencephalons of Tcf4 Knockout (Tcf4-KO) compared to control (WT) mice. The control samples are denoted in green, while the Tcf4 Knockout samples are in red. (**C**) PCA based on the expression of calcium-target signature genes in dorsal telencephalons of Tcf4 Heterozygous (Tcf4-Het) compared to control (WT) mice. The control samples are denoted in green, while the Tcf4 Knockout samples are in red. (**D**) PCA based on the expression of calcium-regulating signature genes in dorsal telencephalons of Tcf4 Heterozygous (Tcf4-Het) compared to control (WT) mice. The control samples are denoted in green, while the Tcf4 Knockout samples are in red.

PCA based on the previously identified AS signatures of calcium-target and calcium-regulating genes revealed that these signature genes are also good classifiers of PTHS in both Tcf4-knockout and heterozygous mouse models (Figure 3). Unsupervised analysis of differentially expressed genes in telencephalons of Tcf4-KO compared to WT mice revealed the enrichment of the ‘calcium’ GO term (Supplementary Figure S2A,B). Utilizing the CaGeDB database, we found that 15.65% (18 genes) of all differentially expressed genes were calcium-related (Table 2, Supplementary Table S4). 

Performing differential expression analysis on the RNA-seq data of Tcf4 heterozygous mice, we did not find any enrichment of the calcium-related GO terms (Supplementary Figure S2C,D). Nevertheless, 11.54% (9 genes) of all dysregulated genes were from the CaDeGB database of known calcium-associated genes (Table 2, Supplementary Table S5).

#### 2.1.3. Phelan-McDermid Syndrome (GSE150429)

Another important mimic of AS is a chromosome 22q13.3 deletion (Phelan-McDermid) syndrome [31]. Phelan-McDermid syndrome (PMS) patients exhibit clinical symptoms similar to AS, including moderate-to-severe developmental delay with absent or minimal speech, neonatal hypotonia, feeding difficulties in infancy, and mouthing behaviors [31]. *SHANK3* has been implicated as the critical gene in the chromosome 22q13.3 deletion syndrome; however, haploinsufficiency of other genes in the region influences the phenotypic expression and severity of the syndrome [32,33]. 

SHANK3 is a member of the Shank family of proteins that interact with various postsynaptic density (PSD) proteins through their functional domains [34]. Most notably, SHANKs bind to synapse-associated protein 90/postsynaptic density 95-associated protein, which, in turn, binds to PSD95 family proteins to form the PSD95/SAPAP/SHANK postsynaptic complex [35]. Although *SHANK3* is not listed in the CaGeDB database of calcium-related genes, a recent study found SHANK3 directly binds CaMKIIα and the L-type calcium channel and that this interaction is crucial for activating downstream CREB signaling [36].

To examine the applicability of the aforementioned AS calcium-target and calcium-regulating gene signatures in PMS and to study the dysregulation of calcium signaling in PMS in general, we utilized the publicly available RNA sequencing dataset GSE150429 [37]. The RNA-seq data were generated from human-induced pluripotent stem cell-based model (hiPSC-neurons) of PMS by reprogramming peripheral blood samples from individuals with PMS (*n* = 7) and their unaffected siblings (*n* = 6) [37]. The etiology of six out of seven PMS patients included the deletion of chromosomal loci, including the *SHANK3* gene. One patient had a frameshift mutation in the *SHANK3* gene. For each participant, three hiPSC clones were generated and differentiated into forebrain neurons (*n* = 41) for three different time periods: 4 weeks, 6 weeks, and 8 weeks. Due to possible differences in calcium signaling between males and females [38], we separately studied the male and female hiPSC neurons differentiated at three different time points.

**Figure 4:**
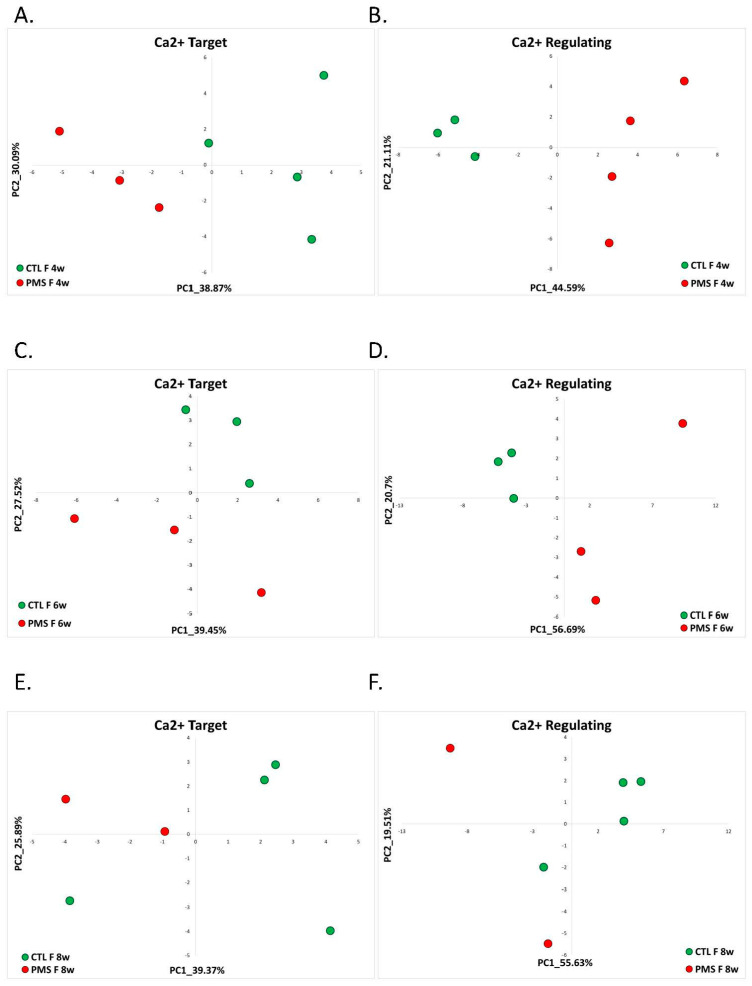

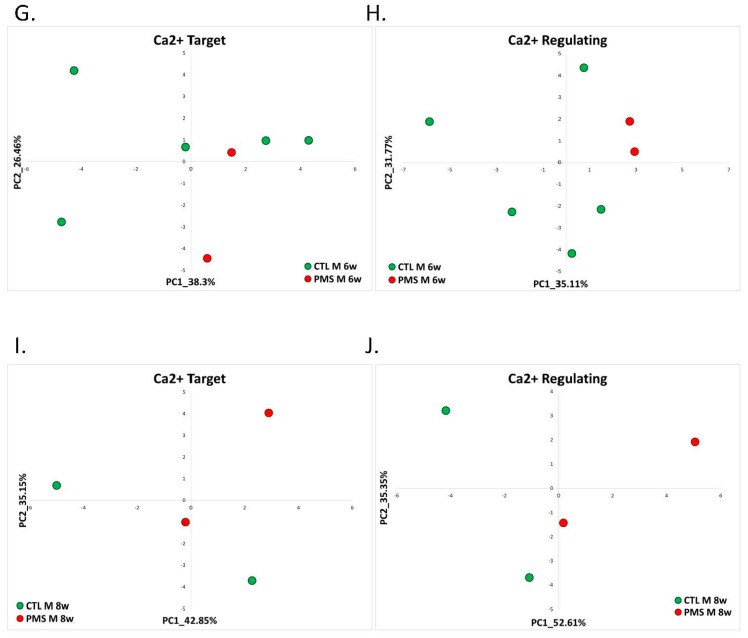
Phelan-McDermid Syndrome: ca-target and ca-regulating signatures (**A**) PCA based on the expression of calcium-target signature genes in hiPSC-neurons differentiated for 4 weeks from female PMS patients and healthy controls. The control hi-PSCs are denoted in green, while the PMS hi-PSCs are in red. (**B**) PCA based on the expression of calcium-regulating signature genes in hiPSC-neurons differentiated for 4 weeks from female PMS patients and healthy controls. The control hi-PSCs are denoted in green, while the PMS hi-PSCs are in red. (**C**) PCA based on the expression of calcium-target signature genes in hiPSC-neurons differentiated for 6 weeks from female PMS patients and healthy controls. The control hi-PSCs are denoted in green, while the PMS hi-PSCs are in red. (**D**) PCA based on the expression of calcium-regulating signature genes in hiPSC-neurons differentiated for 6 weeks from female PMS patients and healthy controls. The control hi-PSCs are denoted in green, while the PMS hi-PSCs are in red. (**E**) PCA based on the expression of calcium-target signature genes in hiPSC-neurons differentiated for 8 weeks from female PMS patients and healthy controls. The control hi-PSCs are denoted in green, while the PMS hi-PSCs are in red. (**F**) PCA based on the expression of calcium-regulating signature genes in hiPSC-neurons differentiated for 8 weeks from female PMS patients and healthy controls. The control hi-PSCs are denoted in green, while the PMS hi-PSCs are in red. (**G**) PCA based on the expression of calcium-target signature genes in hiPSC-neurons differentiated for 6 weeks from male PMS patients and healthy controls. The control hi-PSCs are denoted in green, while the PMS hi-PSCs are in red. (**H**) PCA based on the expression of calcium-regulating signature genes in hiPSC-neurons differentiated for 6 weeks from male PMS patients and healthy controls. The control hi-PSCs are denoted in green, while the PMS hi-PSCs are in red. (**I**) PCA based on the expression of calcium-target signature genes in hiPSC-neurons differentiated for 8 weeks from male PMS patients and healthy controls. The control hi-PSCs are denoted in green, while the PMS hi-PSCs are in red. (**J**) PCA based on the expression of calcium-regulating signature genes in hiPSC-neurons differentiated for 8 weeks from male PMS patients and healthy controls. The control hi-PSCs are denoted in green, while the PMS hi-PSCs are in red.

We found that at all points of neuronal development, the hiPSC neurons derived from female PMS patients were separated from hiPSC neurons derived from female healthy controls on the PC1–PC2 plane based on the expression of previously identified calcium-target and calcium-regulating signature genes (Figure 4A–F). Interestingly, the separation of neurons derived from female PMS patients from hiPSC control samples was less evident at 8 weeks post-differentiation when using the expression of calcium target signature genes (Figure 4E).

In the male samples, we did not consider the hiPSC neurons at the 4-week passaging point because only one PMS sample was sequenced. Interestingly, in males, though the signatures separated between the PMS patients and the healthy controls on the PC1–PC2 plane, this separation was not as pronounced as in females (Figure 4G,H,J). Moreover, the male PMS samples at 8 weeks post-differentiation using calcium target signature also showed a weaker separation of PMS samples from controls (Figure 4I). 

Differential expression analysis did not reveal significantly enriched calcium-associated GO terms in either the female or male hiPSC-derived neurons (Supplementary Figure S3). Utilizing the CaGeDB database of calcium-related genes, we found that in female-derived 4 weeks post-differentiation hiPSC neurons, 11.78% (39 genes, Supplementary Table S6) of all dysregulated genes were calcium-related (Table 2). At 6 weeks post-differentiation female-derived hiPSC, we observed that 11.42% (33 genes, Supplementary Table S7) of all dysregulated genes were calcium-related. Interestingly, at 8 weeks post-differentiation of neurons derived from female PMS patients and healthy controls, we found considerably less differentially expressed genes than at 4 or 6 weeks post-differentiation. Of these dysregulated genes, 8.5% (four genes, Supplementary Table S8) were calcium-related. From male patient-derived hiPSCs, in the 6-week differentiating neurons, we observed 13.4% of all dysregulated genes were calcium-related (41 genes, Supplementary Table S9), and in the 8-week differentiating neurons, 10.96% (40 genes, Supplementary Table S10) of all dysregulated genes were calcium-related. 

#### 2.1.4. Smith-Magenis Syndrome (GSE81206)

Smith-Magenis syndrome (SMS) is a neurobehavioral disorder caused by haploinsufficiency of the retinoic acid-induced 1 (*RAI1*) gene on chromosome 17p11.2 [39]. Unlike Angelman Syndrome that primarily affects the nervous system [40], SMS is a developmental disorder that affects multiple organ systems of the body [41]. SMS patients show a wide range of variability in symptoms. The majority of patients have mild-to-moderate intellectual disability, delayed speech and language skills, distinctive facial features, sleep disturbances, and behavioral problems [42].

Up to date, the *RAI1* gene has been known as a transcriptional factor implicated in cell growth and cell cycle regulation, bone and skeletal development, lipid and glucose metabolisms, embryonic neurodevelopment and neuronal differentiation, behavioral functions, and circadian activity [39]; however, the exact mechanisms of transcript regulation by *RAI1* have not been elucidated yet. In a recent study, Iwase and colleagues [43] have found that *RAI1* is an activity-dependent chromatin remodeler that suppresses synaptic upscaling triggered by activity silencing in the naive network. 

Several mouse models of SMS exist; however, all of these models have severe limitations and do not represent the features of SMS patients well [44,45]. Most Rai1-null mice die in utero, and the few that survive exhibit craniofacial and skeletal abnormalities, motor dysfunction, and fear-learning deficits [44]. Rai1 heterozygous mice display some mild SMS-like symptoms, including obesity, circadian abnormalities, and characteristic craniofacial features [44]. In the herein study of calcium-related transcriptional changes in AS-like syndromes, we did not find a dataset of an SMS model in which Rai1 was deleted from all brain regions. Hence, we studied the publicly available RNA-seq dataset from three brain regions of conditional Rai1 knockdown and Rai1^flox/flox^ control mice (GSE81206) [45]. In these mice, Nestin^Cre^ was used to delete Rai1 from the cortex, Vglut2^Cre^ was used to delete Rai1 from the hypothalamus, and Gad2^Cre^ was used to delete Rai1 from the striatum. 

**Figure 5:**
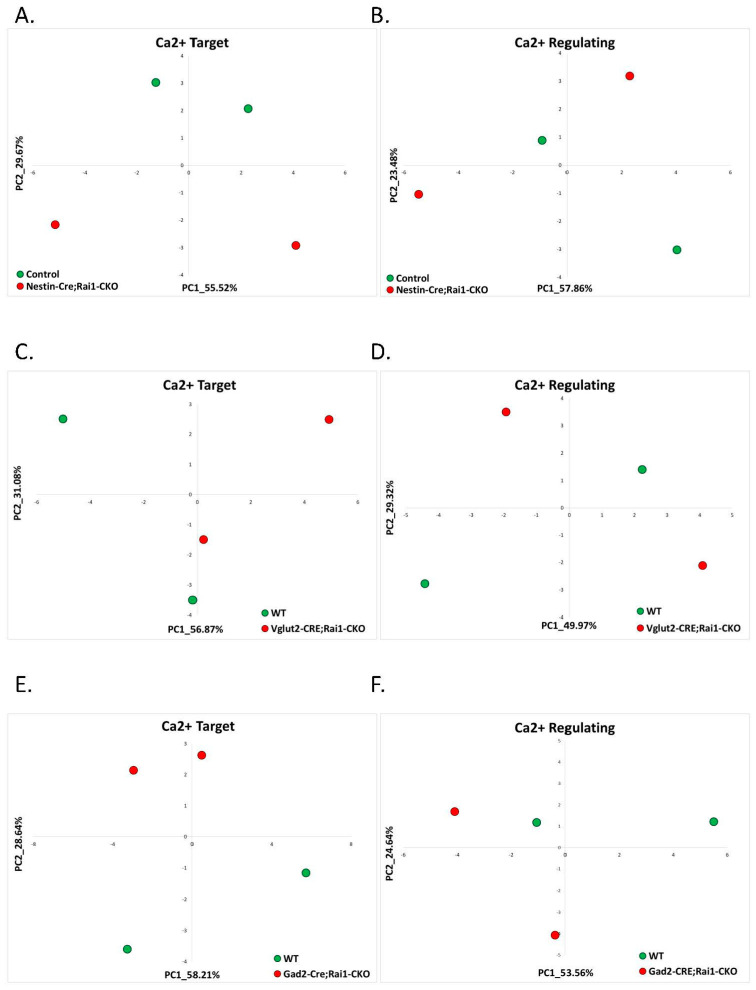

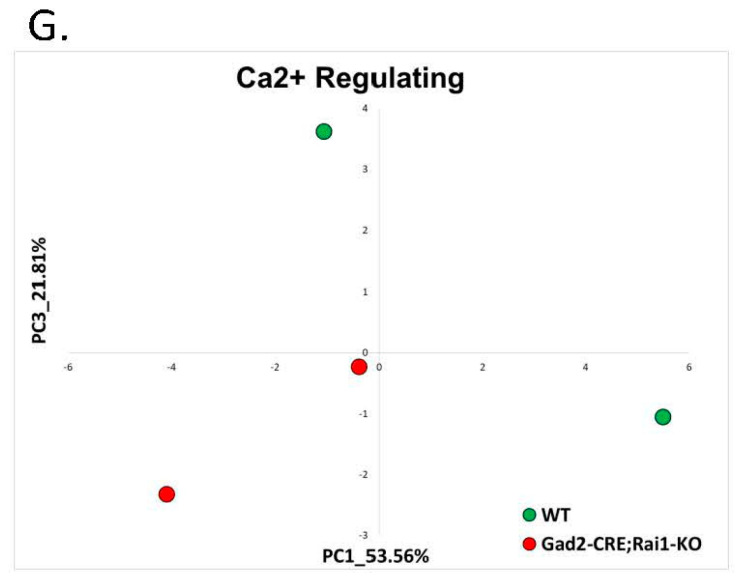
Smith-Magenis Syndrome: calcium target and calcium regulating signatures. (**A**) PCA based on the expression of calcium-target signature genes in the cortices of 3-week-old Nestin^CRE^Rai1^CKO^ (SMS model) mice compared to their controls. The control samples are denoted in green, while the SMS model samples are in red. (**B**) PCA based on the expression of calcium-regulating signature genes in the cortices of 3-week-old Nestin^CRE^Rai1^CKO^ (SMS model) mice compared to their controls. The control samples are denoted in green, while the SMS model samples are in red. (**C**) PCA based on the expression of calcium-target signature genes in the hypothalamus of 8-week-old Vglut2^CRE^Rai1^CKO^ (SMS model) mice compared to their controls. The control samples are denoted in green, while the SMS model samples are in red. (**D**) PCA based on the expression of calcium-regulating signature genes in the hypothalamus of 8-week-old Vglut2^CRE^Rai1^CKO^ (SMS model) mice compared to their controls. The control samples are denoted in green, while the SMS model samples are in red. (**E**) PCA based on the expression of calcium-target signature genes in the striatum of 12-week-old Gad2^CRE^Rai1^CKO^ (SMS model) mice compared to their controls. The control samples are denoted in green, while the SMS model samples are in red. (**F**) PCA based on the expression of calcium-regulating signature genes in the striatum of 12-week-old Gad2^CRE^Rai1^CKO^ (SMS model) mice compared to their controls. The separation of samples on the PC1–PC2 plane. The control samples are denoted in green, while the SMS model samples are in red. (**G**) PCA based on the expression of calcium-regulating signature genes in the striatum of 12-week-old Gad2^CRE^Rai1^CKO^ (SMS model) mice compared to their controls. The separation of samples on the PC1–PC3 plane. The control samples are denoted in green, while the SMS model samples are in red

Nestin^Cre^Rai1^CKO^ mice exhibited postnatal lethality at an increasing frequency with age; thus, only young 3-week-old mice were used to delineate the effect of Rai1 on transcriptome changes in cortical brain tissue. Utilizing the previously found calcium-target and calcium-regulating signatures of AS, we evaluated whether conditional Rai1 knockdown in different brain regions disrupts calcium signaling. In the cortical brain region, the calcium-target signature genes separated the Nestin^Cre^Rai1^CKO^ samples from WT samples well, while the calcium-regulating signature genes were less efficient for Rai1^CKO^ separation (Figure 5A,B). Similarly, in the hypothalamus of Vglut2^Cre^Rai1^CKO^ 8-week-old mice, calcium-target signature genes separated the Rai1^CKO^ mice from their controls, while again, the calcium-regulating signature genes did not (Figure 5C,D). In the striatum of 12-week-old mice, both calcium-target and calcium-regulating signatures separated the Rai1^CKO^ mice from the control WT mice (Figure 5E,F); however, the separation based on calcium-regulating genes was weaker than based on calcium-target genes. Nevertheless, both on the PC1–PC2 plane (Figure 5F) and on the PC1–PC3 plane (Figure 5G), the separation of WT and Rai1^CKO^ mice was evident.

Differential gene expression analysis of the above-mentioned datasets from three brain regions and three developmental stages of Rai1^CKO^ and WT mice revealed no genes that were differentially expressed in Rai1^CKO^ compared to WT control samples (*p*-adjusted < 0.01; absolute fold change > 2). 

#### 2.1.5. Mowat-Wilson Syndrome (GSE84098)

Another single gene syndrome, which is sometimes included in the Angelman-like group of syndromes, is Mowat-Wilson Syndrome (MOWS). Mowat–Wilson syndrome is characterized by moderate-to-severe intellectual disability, distinctive facial appearance, and epilepsy. Unlike Angelman syndrome patients, patients with MOWS develop multiple congenital anomalies, including congenital heart disease, agenesis of the corpus callosum, and eye defects [46]. 

Mowat-Wilson is caused by deleterious de novo heterozygous variations in the *Zeb2* gene that usually cause haploinsufficiency of the Zeb2 protein. The Zeb2 protein, also known as ZFHX1B (zinc finger homeobox 1B) or SIP1 (Smad-interacting protein 1) [47], is a member of the ZEB family of zinc finger transcription factors, which are essential during normal embryonic development [48]. Earlier, it has been shown that Zeb2 regulated calcium signaling pathways at least in the heart [49] and during cerebellar development at P0 [50].

**Figure 6:**
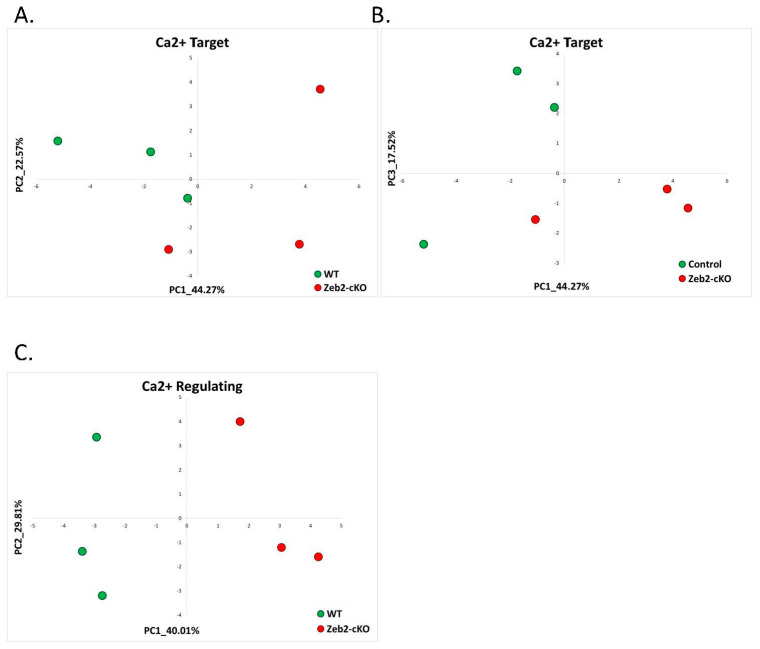
Mowat-Wilson Syndrome: calcium target and calcium regulating signatures. (**A**) PCA based on the expression of calcium-target signature genes in the cerebellum of Zeb2-cKO (deletion of the Zeb2 gene in cerebellar neural progenitors) mice right after birth (P0) and their wild-type (WT) controls. The separation of samples on the PC1–PC2 plane. The control samples are denoted in green, while the Zeb2-cKO samples are in red. (**B**) PCA based on the expression of calcium-target signature genes in the cerebellum of Zeb2-cKO (deletion of the Zeb2 gene in cerebellar neural progenitors) mice right after birth (P0) and their wild-type (WT) controls. The separation of samples on the PC1–PC3 plane. The control samples are denoted in green, while the Zeb2-cKO samples are in red. (**C**) PCA based on the expression of calcium-regulating signature genes in the cerebellum of Zeb2-cKO (deletion of the Zeb2 gene in cerebellar neural progenitors) mice right after birth (P0) and their wild-type (WT) controls. The control samples are denoted in green, while the Zeb2-cKO samples are in red.

To investigate whether the previously identified Angelman Syndrome derived signatures can also be useful in identifying Mowat-Wilson Syndrome, we utilized publicly available RNA-seq data from the cerebellum of Zeb2-cKO (deletion of the Zeb2 gene in cerebellar neural progenitors) mice right after birth (P0) and their WT controls (GSE84098) [50]. Zeb2-cKO mice were bred from Zeb2-floxed mice with a human GFAP promoter-driven Cre (hGFAP-Cre), which is expressed in radial glia or neural stem/progenitors that can give rise to the majority of the cell types, including Bergmann glia, astrocytes, oligodendrocytes, and granule neurons in the cerebellum. We found that based on both calcium-target and calcium-regulating signatures, Zeb2-cKO cerebella were separated from WT controls (Figure 6). Zeb2-cKO mice were clearly separated from controls based on calcium-regulating signature genes (Figure 6C). The calcium-target signature genes also separated Zeb2-cKO mice from controls on both the PC1–PC2 plane (Figure 6A) and the PC1–PC3 plane (Figure 6B).

Differential gene expression analysis did not reveal significantly enriched calcium-associated processes (Supplementary Figure S4A,B). In addition, none of the known calcium-related genes from the CaGeDB database were differentially expressed in Zeb2-cKO mice compared to controls. Nevertheless, earlier analysis by the authors of the dataset showed enrichment of calcium-signaling pathways by upregulated genes in Zeb2-cKO samples [50]. 

### 2.2. Non-AS Like Syndromes

In order to evaluate whether the previously found calcium-target and calcium-regulating signatures are indicators of an AS-like phenotype and not general predictors of neurodevelopmental disorders, we studied several syndromes that are not considered AS-like based on their phenotypic presentation. These syndromes include Fragile X Syndrome, Kabuki Syndrome, and Tuberous Sclerosis. In all of these syndromes, we found that the calcium-target and calcium-regulating signatures found in AS were not good classifiers of these disorders.

#### 2.2.1. Fragile X Syndrome (GSE117248)

Like any neurodevelopmental disorder, Fragile X syndrome entails some similarities with AS, such as intellectual disability, speech impairment, abnormal social and emotional interactions, and autism [51,52]. Nonetheless, there are quite a few differences between the two syndromes. FXS patients have a lower percentage of epileptiform activity (~10%–20%) [53], and they do not have the absence of speech despite having speech impairments. Moreover, Fragile X Syndrome is not limited to Central Nervous System (CNS) and impacts the whole body, including heart disorders, connective tissue dysregulation, gastrointestinal disorders, and mood disorders [54].

Nearly all cases of FXS result from the expansion of CGG repeats in the 5′ UTR region of the X-linked fragile X mental retardation 1 gene (*FMR1*) [52]. The expansion of the CGG triplet to more than 200 copies leads to DNA methylation and thus to transcriptional inactivation and the loss of the *FMR1* gene product known as FMRP (fragile X mental retardation protein) [52]. FMRP is an mRNA binding protein that is involved in activity-dependent translation. FMRP is primarily a translational inhibitor; however, changes in transcription or in RNA stability may also lead to increased protein levels [55]. Recently, it has been shown that FMRP regulates post-transcriptional modifications as well as chromatin organization and modifications [55,56].

To elucidate whether previously found AS calcium-target and calcium-regulating signatures are valid for classifying the non-AS-like syndrome FXS, we utilized a publicly available RNA-seq dataset generated from an isogenic human pluripotent stem cell model (GSE117248) [57]. The neurons were differentiated from human neural progenitor cell-derived hiPSCs, with a midbrain-patterning differentiation protocol using CRISPR/Cas9 to introduce indels in exon 3 of *FMR1*, resulting in a complete loss of FMRP expression.

**Figure 7:**
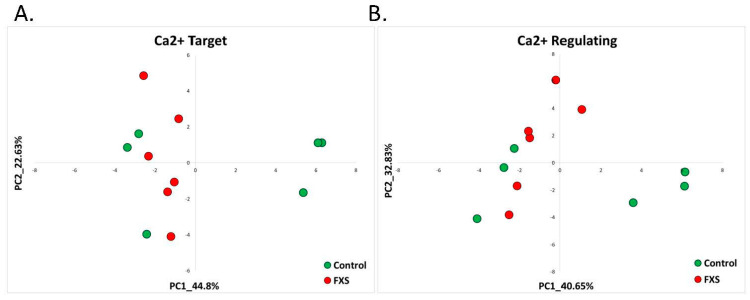
Fragile X Syndrome: calcium-target and calcium-regulating signatures. (**A**) PCA based on the expression of calcium-target signature genes in hiPSCs derived from FXS patients compared to control hiPSCs. The control samples are denoted in green, while the FXS samples are in red. (**B**) PCA based on the expression of calcium-regulating signature genes in hiPSCs derived from FXS patients compared to control hiPSCs. The control samples are denoted in green, while the FXS samples are in red.

Unlike most AS-like syndrome models, PCA based on the above-mentioned AS signatures of calcium-target and calcium-regulating genes did not separate the FXS-model neurons from the control neurons (Figure 7A,B). 

Nonetheless, differential gene expression analysis showed that the ‘calcium-binding’ GO term was enriched by dysregulated genes in FXS-model neurons but not other calcium-related GO terms (Supplementary Figure S5A,B). Utilizing the CaGeDB database of calcium-related genes, we found that in FXS-model neurons, 10.07% (44 genes, Supplementary Table S11) of all dysregulated genes were calcium-related (Table 2).

#### 2.2.2. Kabuki Syndrome (GSE81251)

Kabuki Syndrome (KS) is an additional neurodevelopmental disorder that has a different clinical presentation from AS and is considered a non-AS-like syndrome. KS is characterized by distinct facial dimorphism, growth retardation, psychomotor developmental delay, and a wide spectrum of other manifestations affecting various body systems [58]. KS is a result of a loss of function of either KMT2D or KDM6A proteins. Both of these proteins are histone modifiers that contribute to the opening of chromatin for gene transcription. KMT2D catalyzes the addition of methyl groups to lysine 4 of histone 3 (H3K4me1 and H3K4me3). Both H3K4me1 and H3K4me3 are marks associated with open chromatin; thus, the dysregulation of KMT2D leads to excessive gene transcription. KDM6A also participates in chromatin opening for transcription by removing H3K27me3, which is a mark of closed chromatin [59]. Both genes play a critical role in early vertebrate development, and their reduced expression results in craniofacial, cardiac, and brain abnormalities [58,59,60].

We utilized publicly available RNA-seq data from brains of a neuron-specific Kdm6a-deficient mouse model (GSE81251) to identify whether the aforementioned calcium-target and calcium-regulating signatures could classify the non-AS-like Kabuki Syndrome. 

**Figure 8:**
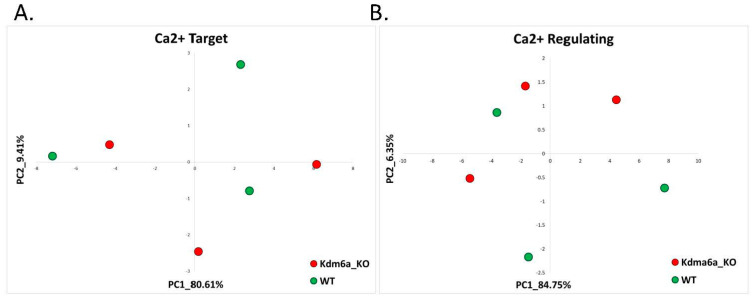
Kabuki Syndrome: calcium-target and calcium-regulating signatures. (**A**) PCA based on the expression of calcium-target signature genes in brain tissue of neuron-specific Kdm6a-deficient mice and their WT controls. The control samples are denoted in green, while the Kdm6a-KO samples are in red. (**B**) PCA based on the expression of calcium-regulating signature genes in brain tissue of neuron-specific Kdm6a-deficient mice and their WT controls. The control samples are denoted in green, while the Kdm6a-KO samples are in red. Differential gene expression analysis revealed no differentially expressed genes in this Kabuki mouse model. Nonetheless, when the threshold of significance was reduced to include only adjusted *p*-values < 0.01 (without any threshold of fold change), the analysis of differentially expressed genes showed that ‘Calcium’ and ‘Calcium-dependent membrane-targeting’ GO terms were significantly enriched (Supplementary Figure S6A,B).

As with the previous non-AS-like syndrome FXS and contrary to the AS-like syndrome models, PCA based on the calcium-target and calcium-regulating gene signatures did not separate the Kabuki mouse model from their control mice littermates on the PC1–PC2 plane (Figure 8A,B). 

#### 2.2.3. Tuberous Sclerosis (GSE78959)

Tuberous Sclerosis (TSC) is a genetic disorder that involves numerous tissues. Apart from the neurological deficits, patients also have heart, kidney, and skin lesions. The neurological symptoms include developmental delay or mental retardation, autism, behavioral problems, and seizures that occur in an overwhelming percentage of TSC patients [61,62].

The two genes that are involved in the etiology of TSC are *TSC1* and *TSC2*, which encode the proteins hamartin and tuberin, respectively. Inactivating mutations in any of these two genes impair the function of the TSC protein complex. This complex acts as an inhibitor of the Ras homolog enriched in brain (Rheb) and mammalian Target of Rapamycin (mTOR) complex 1 (mTORC1) signaling pathway, which controls almost every aspect of cellular metabolism [61,62,63]. 

We utilized a publicly available dataset generated from human neural stem cells derived from embryonic stem cells that have a deletion of the *TSC2* gene (GSE78959) [64]. The dataset includes three cell lines: TSC2^+/+^ (wild-type), TSC2^+/−^ (TSC2-Het), and TSC2^−/−^ (TSC2-KO), which were independently generated from wild-type human embryonic stem cells.

**Figure 9:**
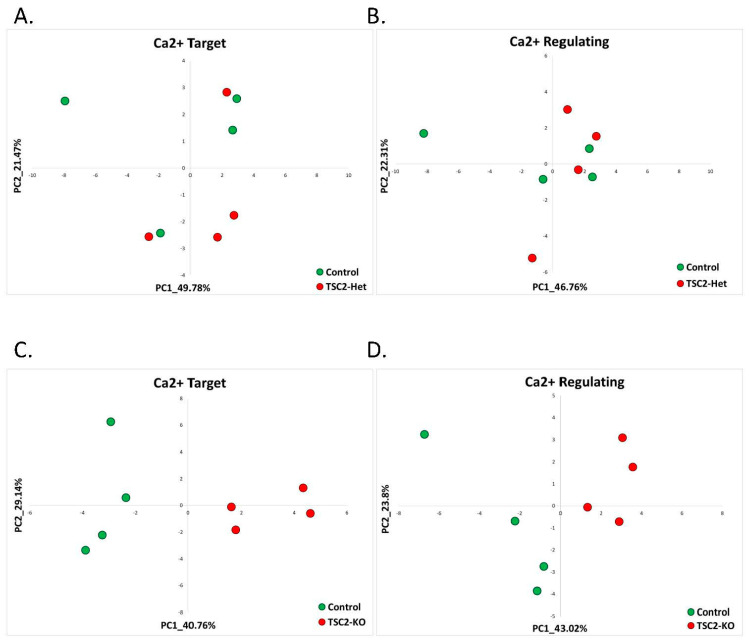
Tuberous sclerosis: calcium-target and calcium-regulating signatures. (**A**) PCA based on the expression of calcium-target signature genes in the TSC2^+/−^ hESCs cell line (TCS2-Het) compared to the control hESCs cell line. The control samples are denoted in green, while the TCS2-Het samples are in red. (**B**) PCA based on the expression of calcium-regulating signature genes in the TSC2^+/−^ hESCs cell line (TCS2-Het) compared to the control hESCs cell line. The control samples are denoted in green, while the TCS2-Het samples are in red. (**C**) PCA based on the expression of calcium-target signature genes in the TSC2^−/−^ hESCs cell line (TCS2-KO) compared to the control hESCs cell line. The control samples are denoted in green, while the TCS2-KO samples are in red. (**D**) PCA based on the expression of calcium-regulating signature genes in the TSC2^−/−^ hESCs cell line (TCS2-KO) compared to the control hESCs cell line. The control samples are denoted in green, while the TCS2-KO samples are in red.

We found that for the TSC2^+/−^ cell line, PCA analyses based on calcium-target and calcium-regulating signatures of AS were not effective in identifying TCS model samples from wild-type controls (Figure 9A,B). Additionally, the differential gene expression analysis did not reveal enriched calcium-associated GO terms (Supplementary Figure S7A,B). Utilizing the CaGeDB database of calcium-related genes, we found that in TSC2^+/−^ cell line, 20% (two genes, Supplementary Table S12) of all dysregulated genes were calcium-related (Table 2).

Interestingly, our previously identified calcium-target and calcium-regulating gene signatures were good classifiers of the cell line with a total deletion of the TSC2 gene (TSC2^−/−^) (Figure 9C,D). It is important to note that TSC patients have a heterozygous deletion and not a complete deletion of either *TSC1* or *TSC2* genes [65]. In addition, unsupervised analysis of differentially expressed genes showed that calcium-associated molecular pathways were enriched by genes dysregulated in TSC2^−/−^ cell lines (Supplementary Figure S7C,D). Based on the CaGeDB database of calcium-related genes, we found that in TSC2^−/−^ cell line, 12.54% (218 genes, Supplementary Table S13) of all dysregulated genes were calcium-related (Table 2).

## 3. Discussion

In a previous study, we found signatures of calcium-target and calcium-regulating genes that perfectly distinguished AS from their controls in different mouse and human models for AS [14]. In a further attempt to investigate the uniqueness of these gene signatures for AS, we herein evaluated whether the calcium-dependent signatures are valid for other neurodevelopmental syndromes. Our initial hypothesis was that these gene signatures were unique for AS. For this aim, we collected RNA-seq datasets of a number of neurodevelopmental syndromes and re-analyzed the transcriptome datasets in light of the calcium-related genes and gene signatures. Surprisingly, we found that calcium-target and calcium-regulating gene signatures were also good classifiers of AS-like syndromes. However, these signatures could not differentiate the neurodevelopmental syndromes with a different clinical presentation from AS and which are not considered AS-like well. This was even more fascinating due to the fact that many of the non-AS-like syndromes had multiple dysregulated calcium-associated genes, as shown by the analyses of differentially expressed genes and their GO term enrichment. Taken together, it is unusual to find that distinctly identified calcium-related gene signatures, which were found using a machine learning approach in several AS models [14], were also able to segregate between other genetic AS-like disorders and their controls. It was even more surprising to find that the calcium-target and calcium-regulating gene signatures were not able to classify neurodevelopmental disorders that do not have a similar clinical presentation to AS. The notion that bioinformatics analyses reflect a general clinical impression towards the categorization of syndromes is quite striking. 

Nonetheless, despite the finding that classification based on calcium-related gene signatures was able to classify five AS-like syndromes but failed to do so with three non-AS-like syndromes, it is important to emphasize that there are plenty more non-AS-like syndromes, and we did not examine this bioinformatics procedure on all syndromes. We chose the ones that we could find a good quality transcriptome with a reasonable disease cellular model. Moreover, the clinical categorization to AS-like and non-AS-like is quite illusive, and it is difficult to clearly delineate between these two categories based on clinical similarity and dissimilarity with AS. Most neurodevelopmental syndromes share many common features, such as intellectual disability, developmental delays, susceptibility for epilepsy, autistic behavior features, and others. Because of that, it would not be surprising if, in the future, this classification would fail for a specific syndrome in a particular model. 

The full meaning of the aforementioned findings is not yet clear, but it puts forward some ideas. For example, it emphasizes the importance of assimilating clinical observations into scientific thinking about the pathophysiological mechanisms of various disorders. In particular, it suggests a specific calcium disruption in AS-like syndromes and points towards a shared final common pathway between these syndromes, thus leading to a similar phenotype. In addition, our findings show that machine-learning approaches applied to available molecular datasets might be useful in the future for the classifications of various disorders. Additionally, the question of whether such findings carry some therapeutic significance is also intriguing. Since if calcium signaling is an important aspect of all AS-like syndromes, then there is a possibility that a pharmacological intervention that will normalize calcium signaling would be beneficial in treating at least some aspects of these disorders. 

## 4. Materials and Methods

Raw expression matrices were downloaded from ncbi GEO collection (https://www.ncbi.nlm.nih.gov/gds/, 11 September 2021). All datasets used in the current study are presented in Table 1. The normalization of raw expression matrices for further downstream analyses was performed with the DeSeq2 algorithm [17]. 

Two lists of calcium-target and calcium-regulating genes (Table 3 and Table 4) that were identified as signatures of AS in our previous study [14] were used to evaluate whether these signatures can be used for other AS-like and non-AS-like neurodevelopmental syndromes. Principle Component Analysis (PCA) [18] of the expression matrices of calcium-target and calcium-regulating signature lists in each dataset was performed in R using the ‘prcomp()’ function.

Differential expression analysis was done with DeSeq2 [17], considering genes with an adjusted *p*-value < 0.01 and an absolute fold change >2 as significantly differentially expressed. DAVID resources [21,66] were used for enrichment analysis of Gene Ontology (GO) terms and biological pathways. Significantly enriched GO terms were considered with a *p*-value < 0.001. The CaGeDB database [15] was utilized to identify calcium-related genes differentially expressed in each disorder compared to its controls.

## 5. Limitations of the Study

The herein study was performed on publicly available datasets collected from NCBI resources. These datasets have a limited number of cases for each disorder, and the models of AS-like disorders include different organisms and different tissues. Nonetheless, despite the fact that the models of AS-like disorders are completely different, they all share the same calcium signatures and could all be identified based on the same gene signatures we determined using AS model mice. This highlights the molecular downstream pathways regulated by *UBE3A* and which are also affected in other AS-like disorders.

## Figures and Tables

**Table 1:** Data sets downloaded from NCBI for analyses.

Disorder	Model	GEO	Number of Samples in Each Dataset
Rett Syndrome	RNA-seq of cerebellum of Mecp2 knockdown male mice and WT controls	GSE105045	RTT = 3 Control = 3
Rett Syndrome	RNA-seq of postmortem brain tissue samples of female patients clinically diagnosed with Rett syndrome and age-matched female donors	GSE128380	Cingulate cortex: RTT = 2 and control = 2. Temporal cortex: RTT = 3 and control = 3
Pitt-Hopkins	RNA sequencing dataset of dorsal telencephalons of Tcf4-knockout, Tcf4-heterozygous, and WT mice at P0 (immediately after birth)	GSE79663	Tcf4-KO = 3; Tcf4-Het = 3; control = 3
Phelan-McDermid Syndrome	RNA-seq data generated from human-induced pluripotent stem cell-based model (hiPSC-neurons) of PMS by reprogramming peripheral blood samples from individuals with PMS (n = 7) and their unaffected siblings (n = 6)	GSE150429	Female 4w: PMS = 3 and control = 4. Female 6w: PMS = 3 and control = 3. Female 8w: PMS = 2 and control = 4. Male 6w: PMS = 2 and control = 5. Male 8w: PMS = 2 and control = 2.
Smith-Magenis Syndrome	RNA-seq of Rai1 conditional knockouts and wild-type mice	GSE81206	Cortex: SMS = 2 and control = 2 Hypothalamus: SMS = 2 and control = 2 Striatum: SMS = 2 and control = 2
Mowat-Wilson Syndrome	RNA-seq data generated from P0 cerebellum of Zeb2-cKO mice and of their WT controls	GSE84098	Zeb-cKO = 3 Control = 3
Fragile X Syndrome	RNA-seq dataset generated from human neural progenitor cells-derived hiPSCs with a midbrain-patterning differentiation protocol using CRISPR/Cas9 to introduce indels in exon 3 of FMR1	GSE117248	FXS = 6 Control = 6
Kabuki Syndrome	RNA-seq data from brains of a neuron-specific Kdm6a-deficient mouse model	GSE81251	Kdma6a-KO = 6 Control = 6
Tuberous Sclerosis	RNA-seq generated from human neural stem cells derived from embryonic stem cells that have a deletion (either TSC2^+/−^ (TSC2-Het) or TSC2^−/−^ (TSC2-KO)) of the TSC2 gene	GSE78959	TSC2-KO = 4 TSC2-Het = 4 Control = 4

**Table 2:** Differentially expressed calcium-target and calcium-regulating genes.

	Disorder	Model	Calcium Target		Calcium Regulating		
			Down Regulated (% from Downregulated)	Up Regulated (% from Upregulated)	Down Regulated (% from Downregulated)	Up Regulated (% from Upregulated)	All Calcium-Related DEG
1	1.1 Rett Syndrome	Mecp2-KO model (GSE105045)	3 (9.37%)	0 (0%)	1 (3.18%)	0 (0%)	12.5%
2		Postmortem brain cingulate cortex (GSE128380)	28 (14.5%)	28 (6.89%)	13 (6.73%)	17 (4.19%)	14.35%
3		Postmortem brain temporal cortex (GSE128380)	46 (10.55%)	63 (7.04%)	33 (7.56%)	35 (3.91%)	13.31%
4	1.2 Pitt-Hopkins	Tcf4-KO (GSE79663)	12 (15%)	1 (2.86%)	5 (6.25%)	0 (0%)	15.65%
5		Tcf4-Het (GSE79663)	4 (6.89%)	0 (0%)	5 (8.62%)	0 (0%)	11.54%
6	1.3 Phelan-McDermid	PMS Female 4w hiPSC neurons (GSE150429)	2 (2.89%)	13 (4.58%)	2 (2.89%)	22 (8.39%)	11.78%
7		PMS Female 6w hiPSC neurons(GSE150429)	2 (1.92%)	14 (7.57%)	3 (2.88%)	14 (7.57%)	11.42%
8		PMS Female 8w hiPSC neurons (GSE150429)	1 (5%)	0 (0%)	0 (0%)	3 (11.11%)	8.5%
9		PMS Male 6w hiPSC neurons (GSE150429)	21 (8.36%)	8 (14.55%)	11 (4.38%)	1 (1.82%)	13.4%
10		PMS Male 8w hiPSC neurons (GSE150429)	14 (5.34%)	12 (11.65%)	12 (4.58%)	2 (1.94%)	10.96%
11	1.4 Smith-Magenis	Rai1-CKO Cortex (GSE81206)	0 (0%)	0 (0%)	0 (0%)	0 (0%)	0%
12		Rai1-CKO Hypothalamus (GSE81206)	0 (0%)	0 (0%)	0 (0%)	0 (0%)	0%
13		Rai1-CKO Striatum (GSE81206)	0 (0%)	0 (0%)	0 (0%)	0 (0%)	0%
14	1.5 Mowat-Wilson	Zeb2-cKO cerebellum (P0) (GSE84098)	0 (0%)	0 (0%)	0 (0%)	0 (0%)	0%
15	2.1 Fragile X	hiPSC indel exon3 FMR1 (GSE117248)	10 (4.33%)	15 (7.28%)	7 (3.03%)	12 (5.82%)	10.07%
16	2.2 Kabuki Syndrome	brains of neuron-specific Kdm6a deficient mice	0 (0%)	0 (0%)	0 (0%)	0 (0%)	0%
17	2.3 Tuberous Sclerosis	human neural stem cells derived from embryonic stem cells TSC2^+/−^ (TSC2-Het)	1 (25%)	1 (16.67%)	0 (0%)	0 (0%)	20%
18		human neural stem cells derived from embryonic stem cell sTSC2^−/−^ (TSC2-KO)	78 (8.76%)	56 (6.59%)	49 (5.50%)	35 (4.12%)	12.54%

**Table 3:** Calcium-target signature genes identified in AS model mice.

GeneSymbol	Ensemble_Mouse	Ensemble_Human
Syt11	ENSMUSG00000068923	ENSG00000132718
Nedd4	ENSMUSG00000032216	ENSG00000069869
Phkb	ENSMUSG00000036879	ENSG00000102893
Braf	ENSMUSG00000002413	ENSG00000157764
Macf1	ENSMUSG00000028649	ENSG00000127603
Ahcyl1	ENSMUSG00000027893	ENSG00000168710
Grm5	ENSMUSG00000049583	ENSG00000168959
Atpif1	ENSMUSG00000054428	ENSG00000130770
Mcfd2	ENSMUSG00000024150	ENSG00000180398
Myo6	ENSMUSG00000033577	ENSG00000196586
Cpne7	ENSMUSG00000034796	ENSG00000178773
Adcy1	ENSMUSG00000020431	ENSG00000164742
Agrn	ENSMUSG00000041936	ENSG00000104490
Ncald	ENSMUSG00000051359	ENSG00000188157
Dgkg	ENSMUSG00000022861	ENSG00000058866
Dst	ENSMUSG00000026131	ENSG00000067715
Syt1	ENSMUSG00000035864	ENSG00000151914
Fus	ENSMUSG00000030795	ENSG00000089280
Chp1	ENSMUSG00000014077	ENSG00000187446
Rbm22	ENSMUSG00000024604	ENSG00000086589
Rab3gap1	ENSMUSG00000036104	ENSG00000115839
Sptan1	ENSMUSG00000057738	ENSG00000197694
Pdcd6ip	ENSMUSG00000032504	ENSG00000170248
Camk1d	ENSMUSG00000039145	ENSG00000183049
Usp32	ENSMUSG00000000804	ENSG00000143622
Rit1	ENSMUSG00000028057	ENSG00000170832
Sparc	ENSMUSG00000018593	ENSG00000113140
Hspa5	ENSMUSG00000026864	ENSG00000044574
Spock1	ENSMUSG00000056222	ENSG00000152377
Ppm1f	ENSMUSG00000026181	ENSG00000100034

**Table 4:** Calcium-regulating signature genes identified in AS model mice.

GeneSymbol	Ensemble_Mouse	Ensemble_human
Cacna1g	ENSMUSG00000020866	ENSG00000006283
Ppp3r1	ENSMUSG00000033953	ENSG00000221823
Fkbp1a	ENSMUSG00000032966	ENSG00000088832
Hsp90b1	ENSMUSG00000020048	ENSG00000166598
Sri	ENSMUSG00000003161	ENSG00000075142
Calm1	ENSMUSG00000001175	ENSG00000198668
Rgs4	ENSMUSG00000038530	ENSG00000117152
Herpud1	ENSMUSG00000031770	ENSG00000051108
Ywhae	ENSMUSG00000020849	ENSG00000108953
Gsto1	ENSMUSG00000025068	ENSG00000148834
Opa1	ENSMUSG00000038084	ENSG00000198836
Bnip3	ENSMUSG00000078566	ENSG00000176171
Nrxn1	ENSMUSG00000024109	ENSG00000179915
Arrb2	ENSMUSG00000060216	ENSG00000141480
Adcy3	ENSMUSG00000020654	ENSG00000138031
Fyn	ENSMUSG00000019843	ENSG00000198947
Dmd	ENSMUSG00000045103	ENSG00000010810
Calr	ENSMUSG00000003814	ENSG00000179218
Slc9a1	ENSMUSG00000028854	ENSG00000090020
Stoml2	ENSMUSG00000028455	ENSG00000165283
Ppp3cb	ENSMUSG00000021816	ENSG00000107758
Ddit3	ENSMUSG00000025408	ENSG00000175197
Stim2	ENSMUSG00000039156	ENSG00000109689
Micu1	ENSMUSG00000020111	ENSG00000107745
Dlg4	ENSMUSG00000020886	ENSG00000132535
Atp13a2	ENSMUSG00000036622	ENSG00000159363
Nptn	ENSMUSG00000032336	ENSG00000156642
Gnb5	ENSMUSG00000032192	ENSG00000069966
Sgk1	ENSMUSG00000019970	ENSG00000118515
Tpt1	ENSMUSG00000060126	ENSG00000133112

## Data Availability

All datasets used for the study are reported in Table 1 of this manuscript.

## Supplementary Materials

The following are available online at https://www.mdpi.com/article/10.3390/ijms22189870/s1.
