# Supplementary figures and images for: Angelman Syndrome and Angelman-like Syndromes Share the Same Calcium-Related Gene Signatures
Source: Int J Mol Sci. 2021 Sep 13;22(18):9870. doi: 10.3390/ijms22189870 (PMC8469403; doi:10.3390/ijms22189870)

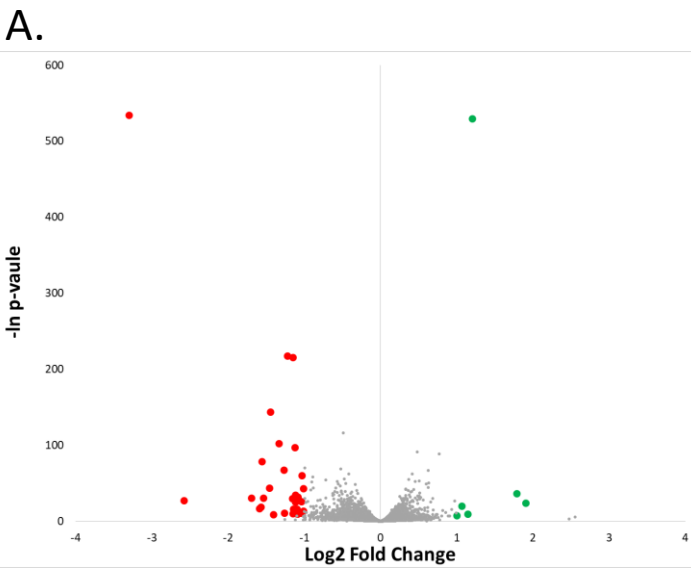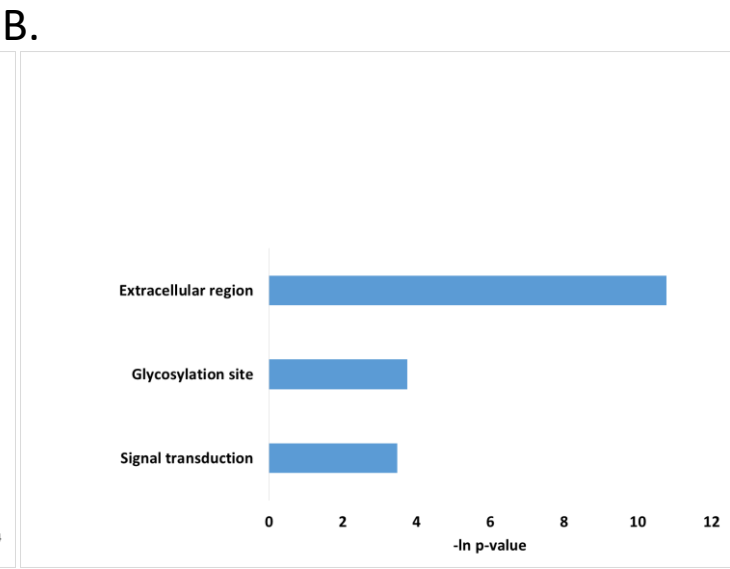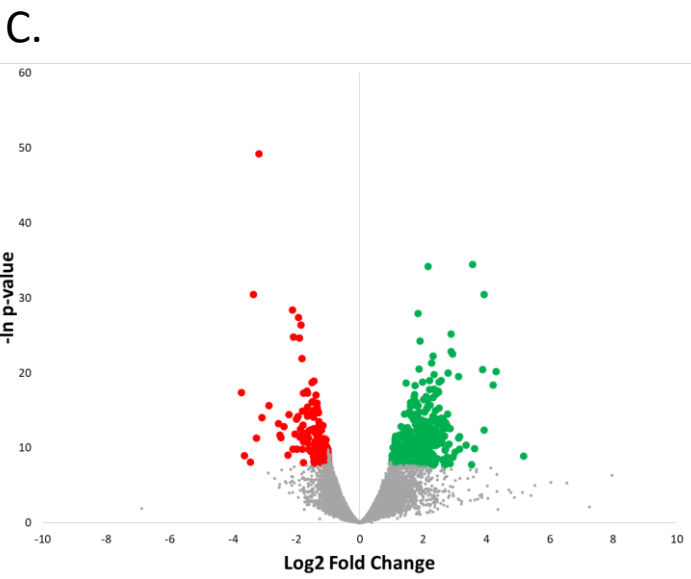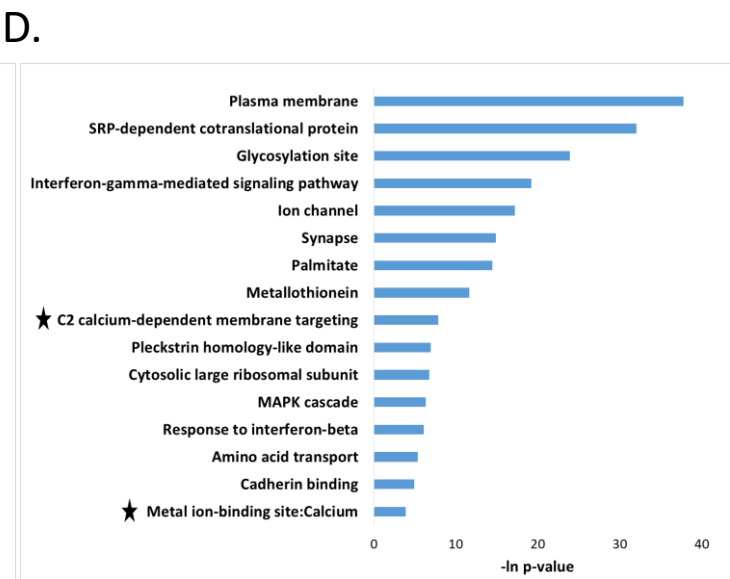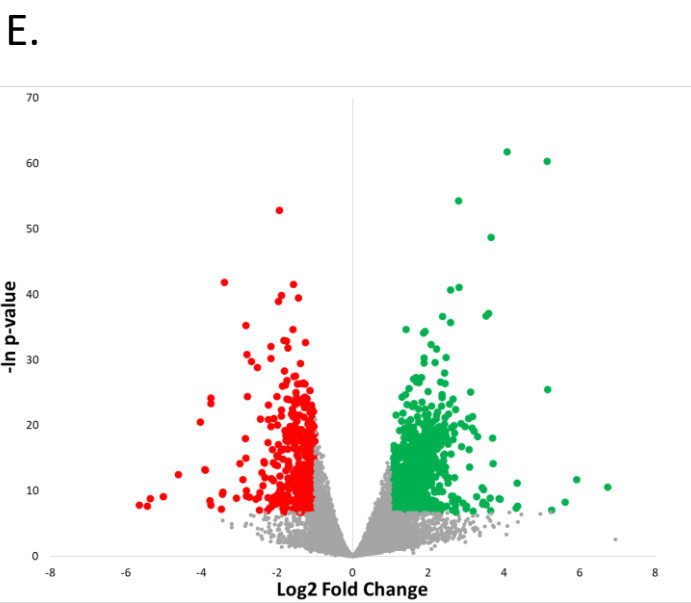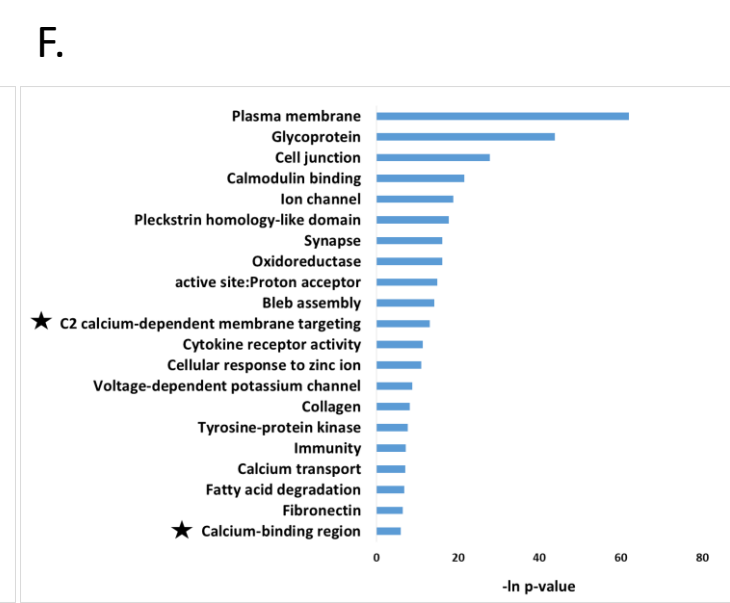

Supplement: Supplementary file 1 [file ijms-22-09870-s001.zip › SupplementaryFigure1_sept3.pdf]

A.

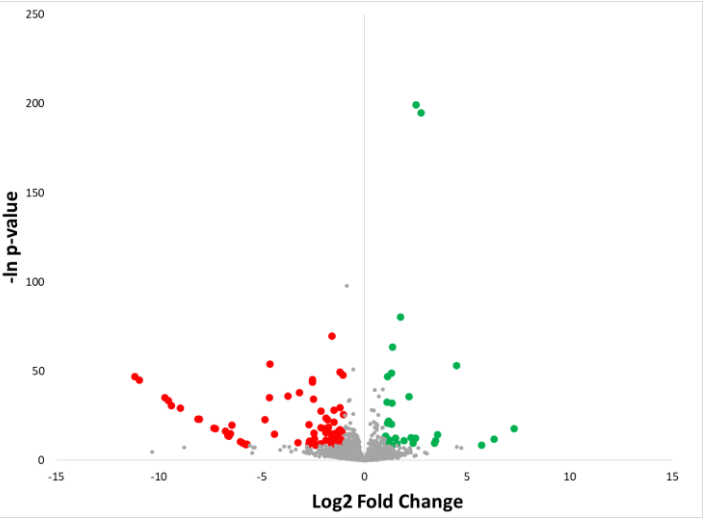

B.

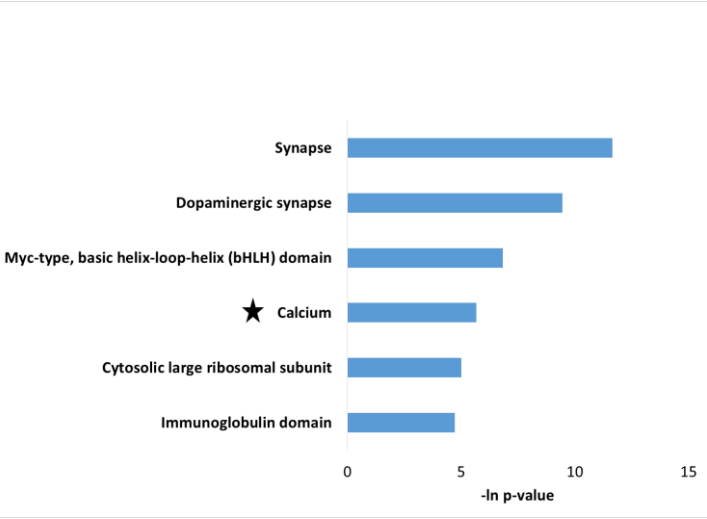

C.

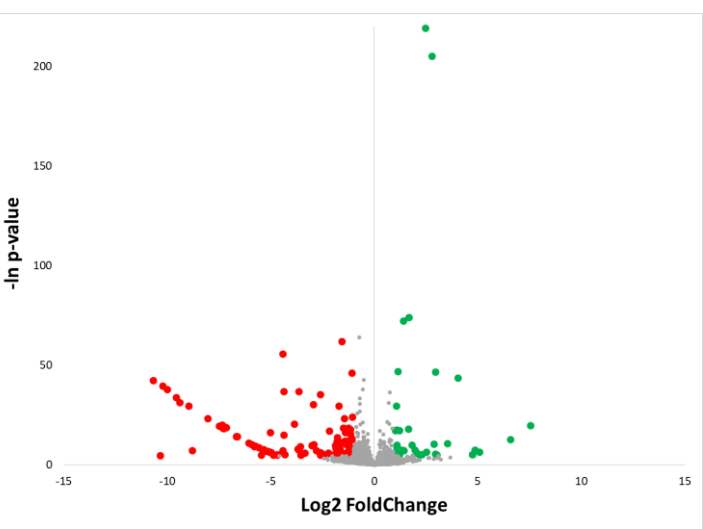

D.

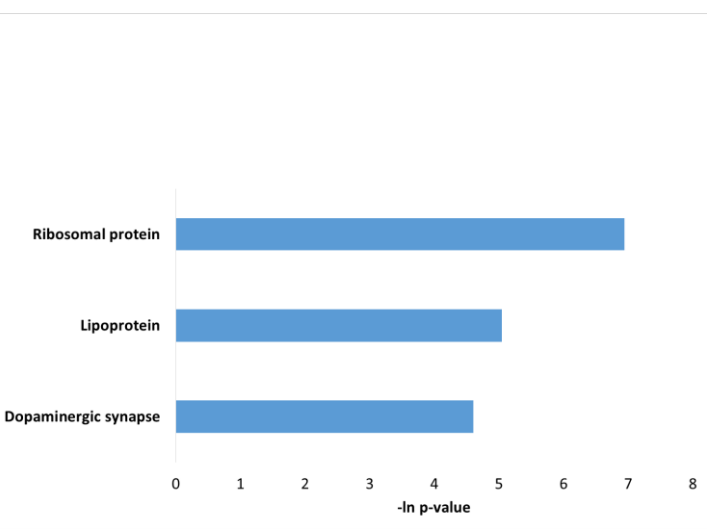

Supplement: Supplementary file 1 [file ijms-22-09870-s001.zip › SupplementaryFigure2_sept3.pdf]

A.

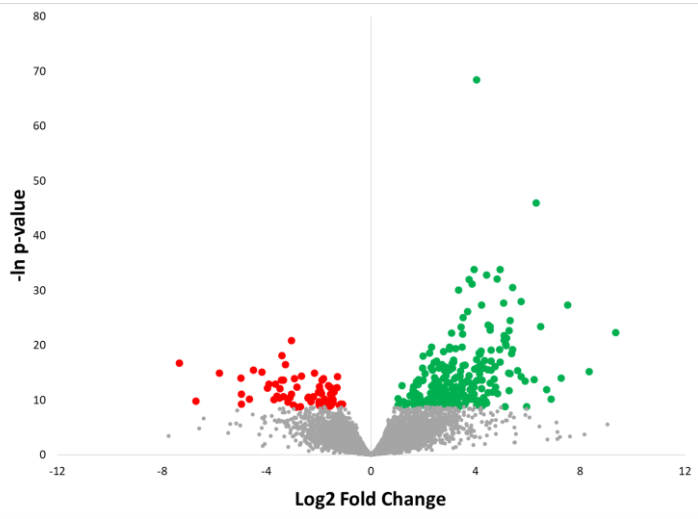

B.

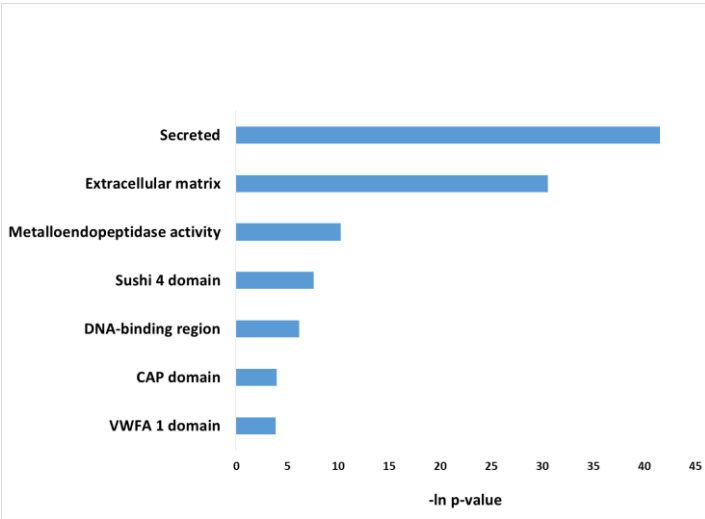

C.

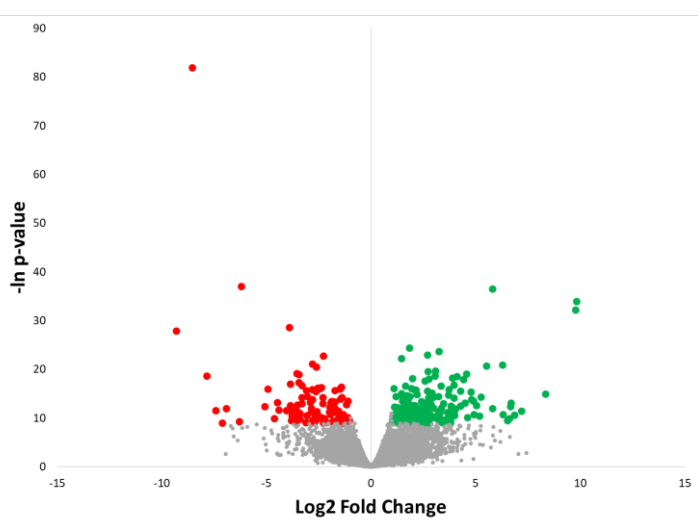

D.

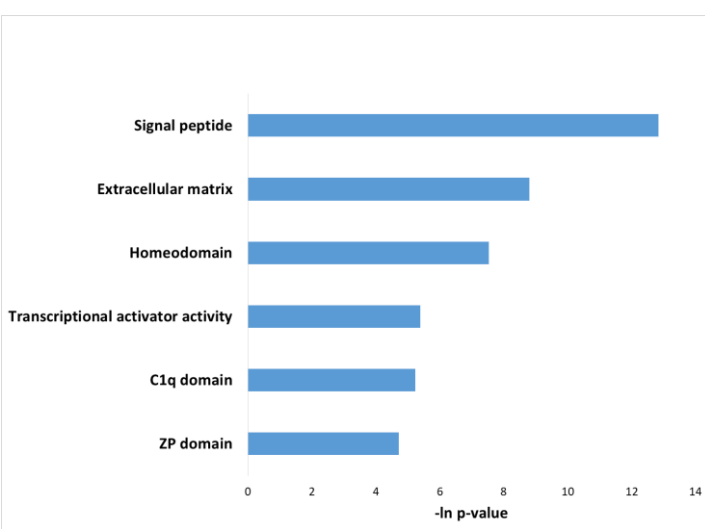

E.

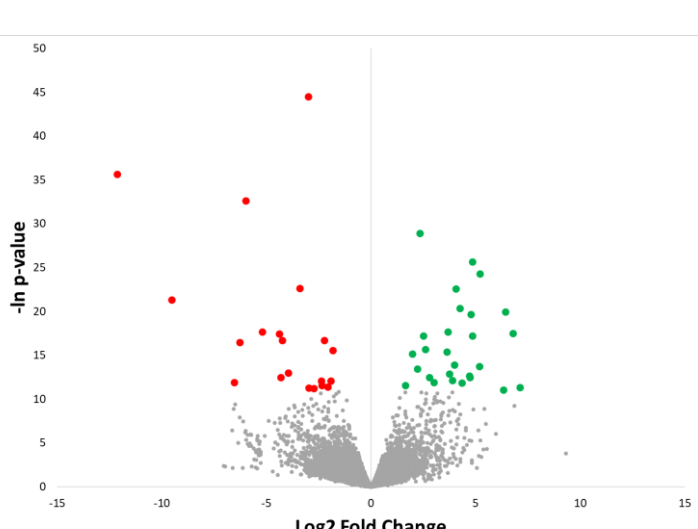

F.

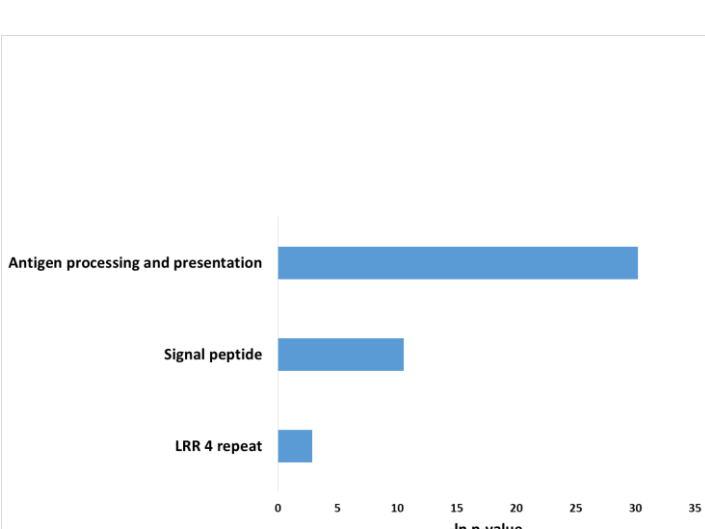

G.

H.

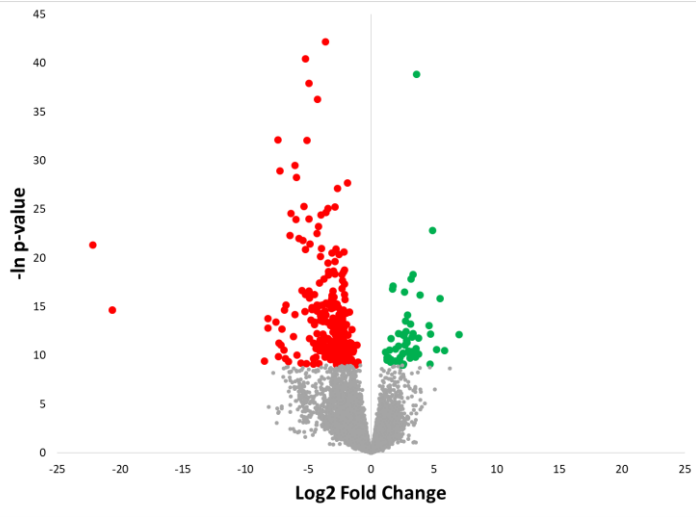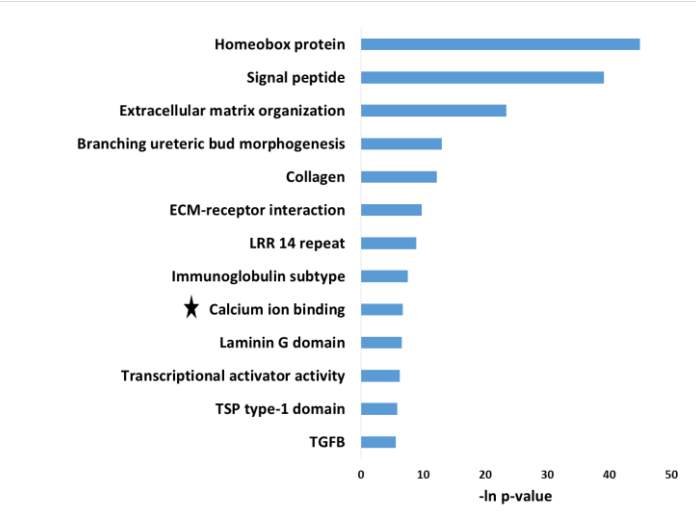

I.

J.

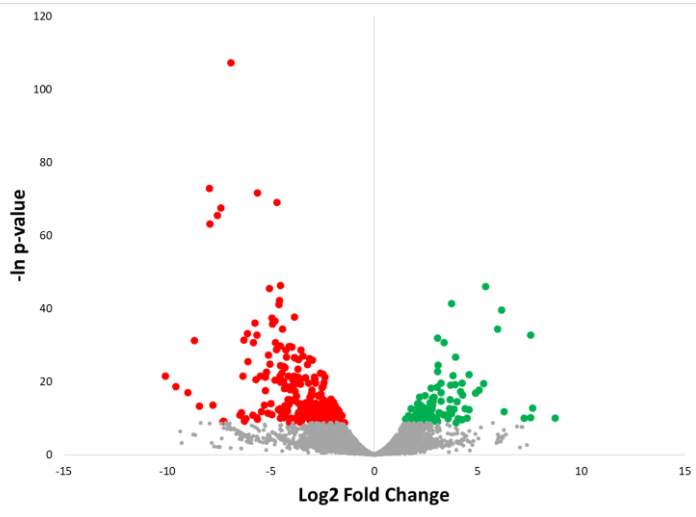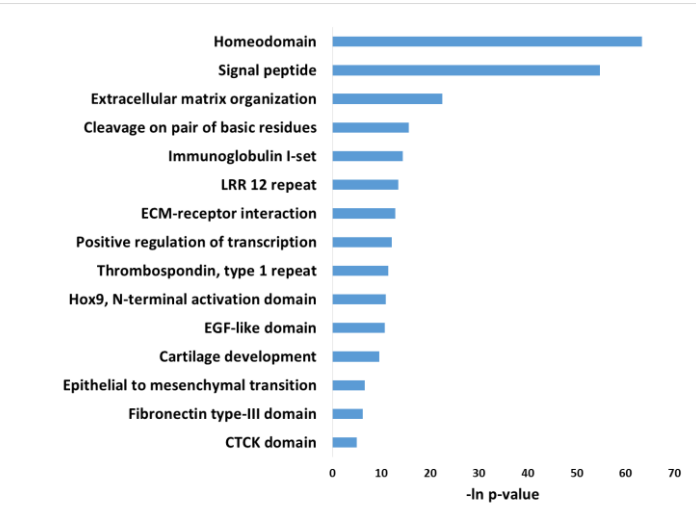

Supplement: Supplementary file 1 [file ijms-22-09870-s001.zip › SupplementaryFigure3_sept3.pdf]

A.

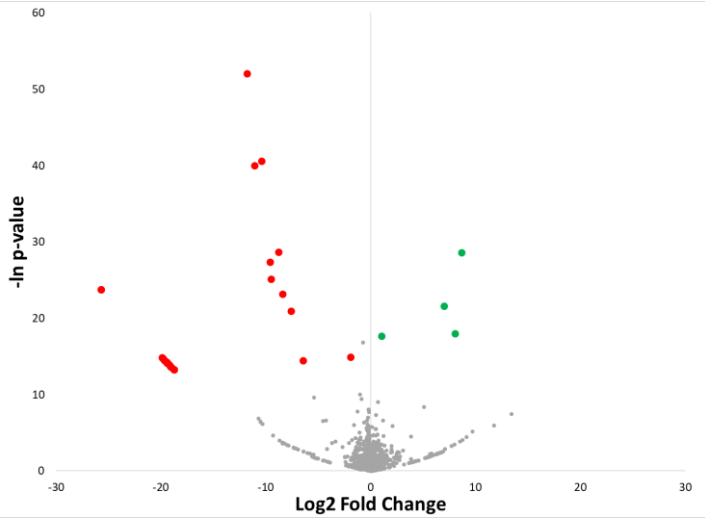

B.

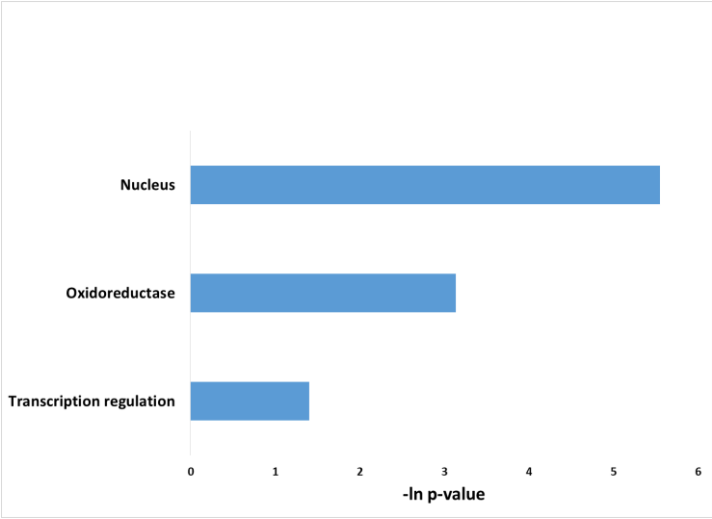

Supplement: Supplementary file 1 [file ijms-22-09870-s001.zip › SupplementaryFigure4_sept3.pdf]

A.

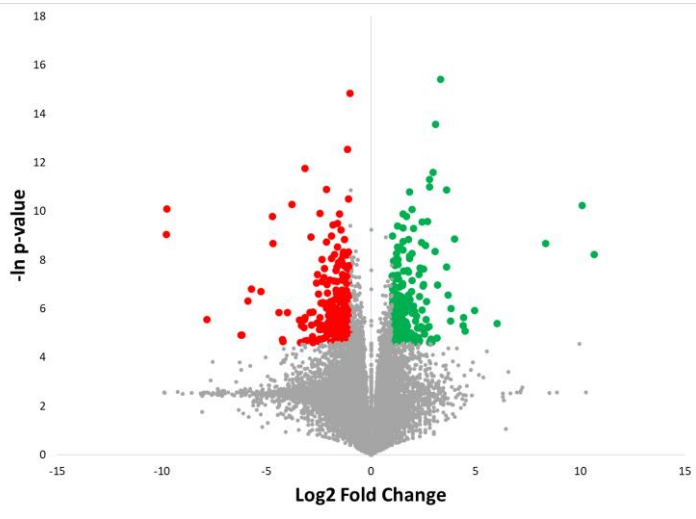

B.

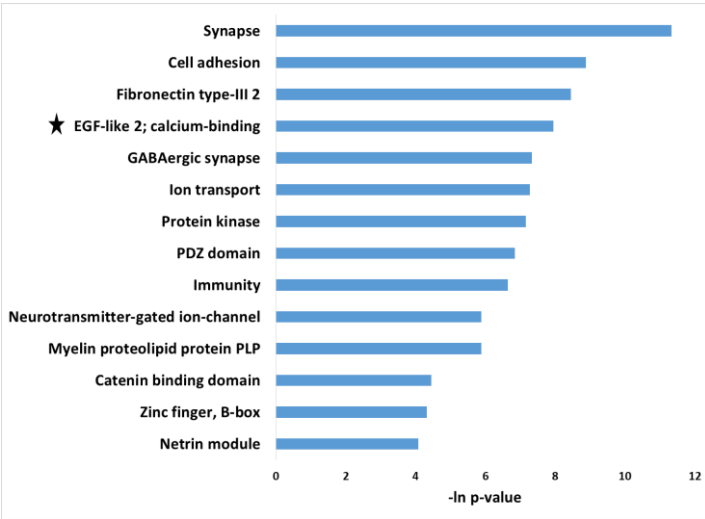

Supplement: Supplementary file 1 [file ijms-22-09870-s001.zip › SupplementaryFigure5_sept3.pdf]

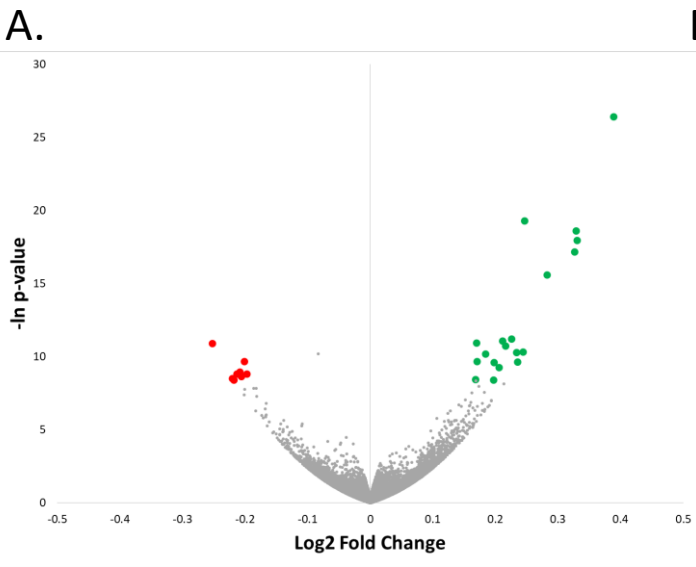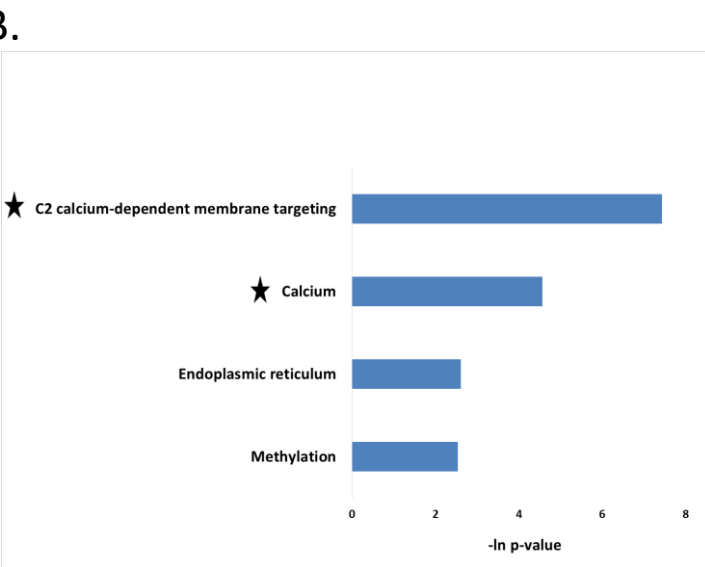

Supplement: Supplementary file 1 [file ijms-22-09870-s001.zip › SupplementaryFigure6_sept3.pdf]

A.

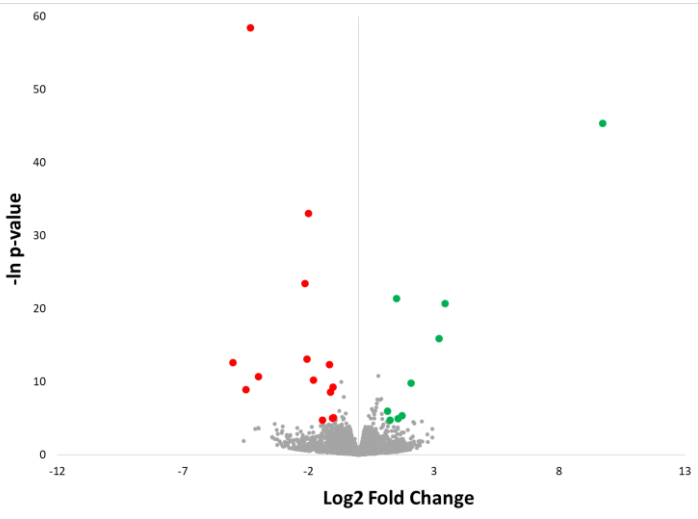

B.

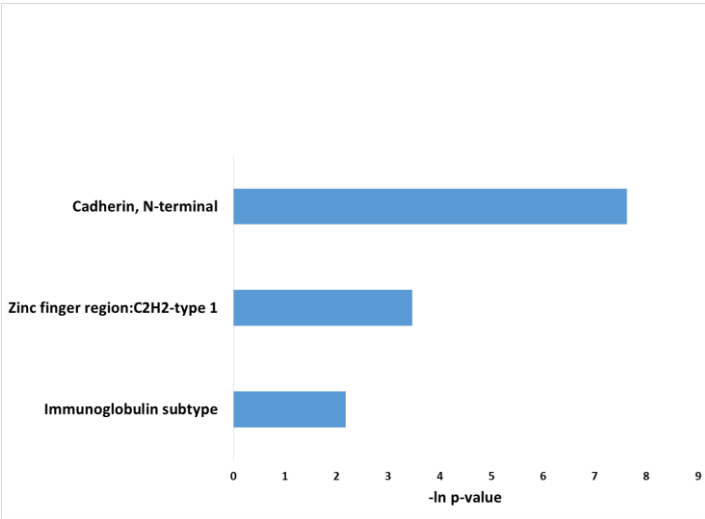

C.

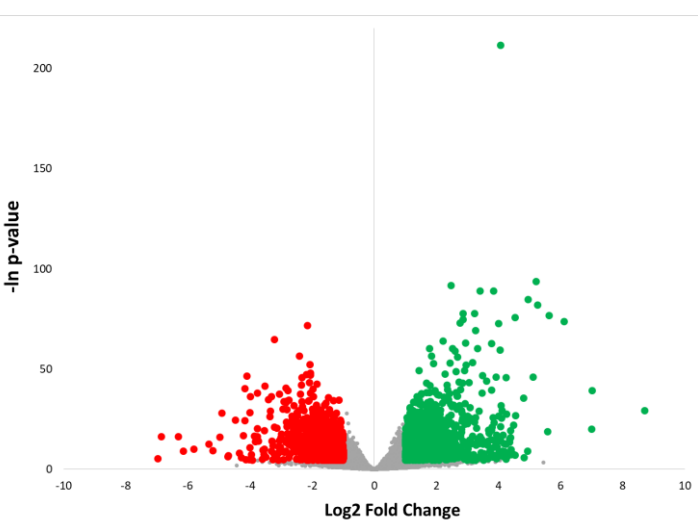

D.

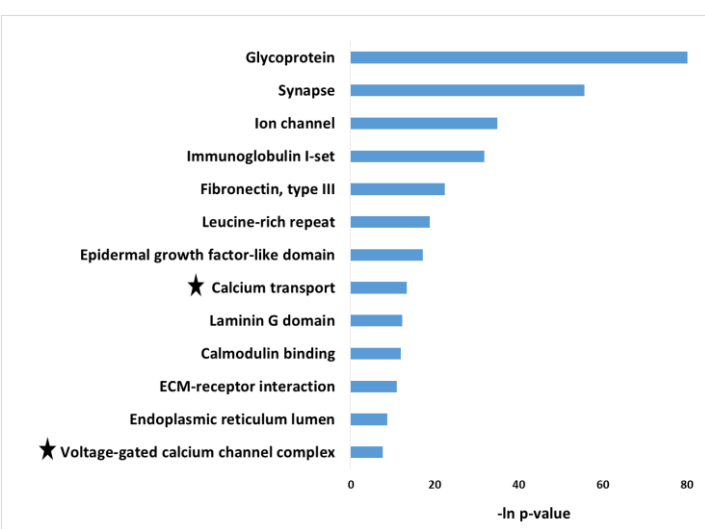

Supplement: Supplementary file 1 [file ijms-22-09870-s001.zip › SupplementaryFigure7_sept3.pdf]
